# A Taz1- and Microtubule-Dependent Regulatory Relationship between Telomere and Centromere Positions in Bouquet Formation Secures Proper Meiotic Divisions

**DOI:** 10.1371/journal.pgen.1006304

**Published:** 2016-09-09

**Authors:** Kazuhiro Katsumata, Ami Hirayasu, Junpei Miyoshi, Eriko Nishi, Kento Ichikawa, Kazuki Tateho, Airi Wakuda, Hirotada Matsuhara, Ayumu Yamamoto

**Affiliations:** 1 Graduate School of Integrated Science and Technology, Shizuoka University, Shizuoka, Japan; 2 Faculty of Science, Shizuoka University, Shizuoka, Japan; 3 Graduate School of Science and Technology, Shizuoka University, Shizuoka, Japan; Stowers Institute for Medical Research, UNITED STATES

## Abstract

During meiotic prophase, telomeres cluster, forming the bouquet chromosome arrangement, and facilitate homologous chromosome pairing. In fission yeast, bouquet formation requires switching of telomere and centromere positions. Centromeres are located at the spindle pole body (SPB) during mitotic interphase, and upon entering meiosis, telomeres cluster at the SPB, followed by centromere detachment from the SPB. Telomere clustering depends on the formation of the microtubule-organizing center at telomeres by the linker of nucleoskeleton and cytoskeleton complex (LINC), while centromere detachment depends on disassembly of kinetochores, which induces meiotic centromere formation. However, how the switching of telomere and centromere positions occurs during bouquet formation is not fully understood. Here, we show that, when impaired telomere interaction with the LINC or microtubule disruption inhibited telomere clustering, kinetochore disassembly-dependent centromere detachment and accompanying meiotic centromere formation were also inhibited. Efficient centromere detachment required telomere clustering-dependent SPB recruitment of a conserved telomere component, Taz1, and microtubules. Furthermore, when artificial SPB recruitment of Taz1 induced centromere detachment in telomere clustering-defective cells, spindle formation was impaired. Thus, detachment of centromeres from the SPB without telomere clustering causes spindle impairment. These findings establish novel regulatory mechanisms, which prevent concurrent detachment of telomeres and centromeres from the SPB during bouquet formation and secure proper meiotic divisions.

## Author Summary

Meiosis is a type of cell division, that generates haploid gametes and is essential for sexual reproduction. During meiosis, telomeres cluster on a small region of the nuclear periphery, forming a conserved chromosome arrangement referred to as the “bouquet”. Because the bouquet arrangement facilitates homologous chromosome pairing, which is essential for proper meiotic chromosome segregation, it is of great importance to understand how the bouquet arrangement is formed. In fission yeast, the bouquet arrangement requires switching of telomere and centromere positions. During mitosis, centromeres are located at the fungal centrosome called the spindle pole body (SPB). Upon entering meiosis, telomeres cluster at the SPB, and centromeres become detached from the SPB, forming the bouquet arrangement. In this study, we show that centromere detachment is linked with telomere clustering. When telomere clustering was inhibited, centromere detachment was also inhibited. This regulatory relationship depended on a conserved telomere component, Taz1, and microtubules. Furthermore, we show that the regulatory relationship is crucial for proper meiotic divisions when telomere clustering is defective. Our findings reveal a hitherto unknown regulatory relationship between meiotic telomere and centromere positions in bouquet formation, which secures proper meiotic divisions.

## Introduction

Chromosome positioning changes dynamically during development and differentiation, and contributes to various chromosomal events including gene expression and DNA metabolism [1–5]. Especially during meiosis, chromosomes adopt a characteristic position called the “bouquet” arrangement, in which telomeres cluster at the nuclear periphery. The bouquet arrangement is highly conserved among eukaryotes [6, 7], and how it is formed and what functions it has are important questions in the field of meiosis.

Studies of various organisms show that the bouquet arrangement facilitates homologous chromosome pairing [7–9]. Bouquet-defective mutants of yeasts and mammals exhibit impaired homologous chromosome pairing and phenotypes associated with the impaired pairing, such as increased non-homologous association, decreased recombination, and defective formation of the synaptonemal complex, a structure that bridges the paired homologous chromosomes [10–23]. In *Caenorhabditis elegans*, special chromosome regions called “pairing centers” cluster instead of telomeres, and impaired clustering of pairing centers causes similar defects [24–29]. Homologous chromosome pairing is essential for formation of chiasmata, which physically link homologous chromosomes and enable their segregation at meiosis I (reductional segregation). Consistently, bouquet-defective organisms also exhibit impaired chromosome segregation [10, 14, 30].

**Fig 1:**
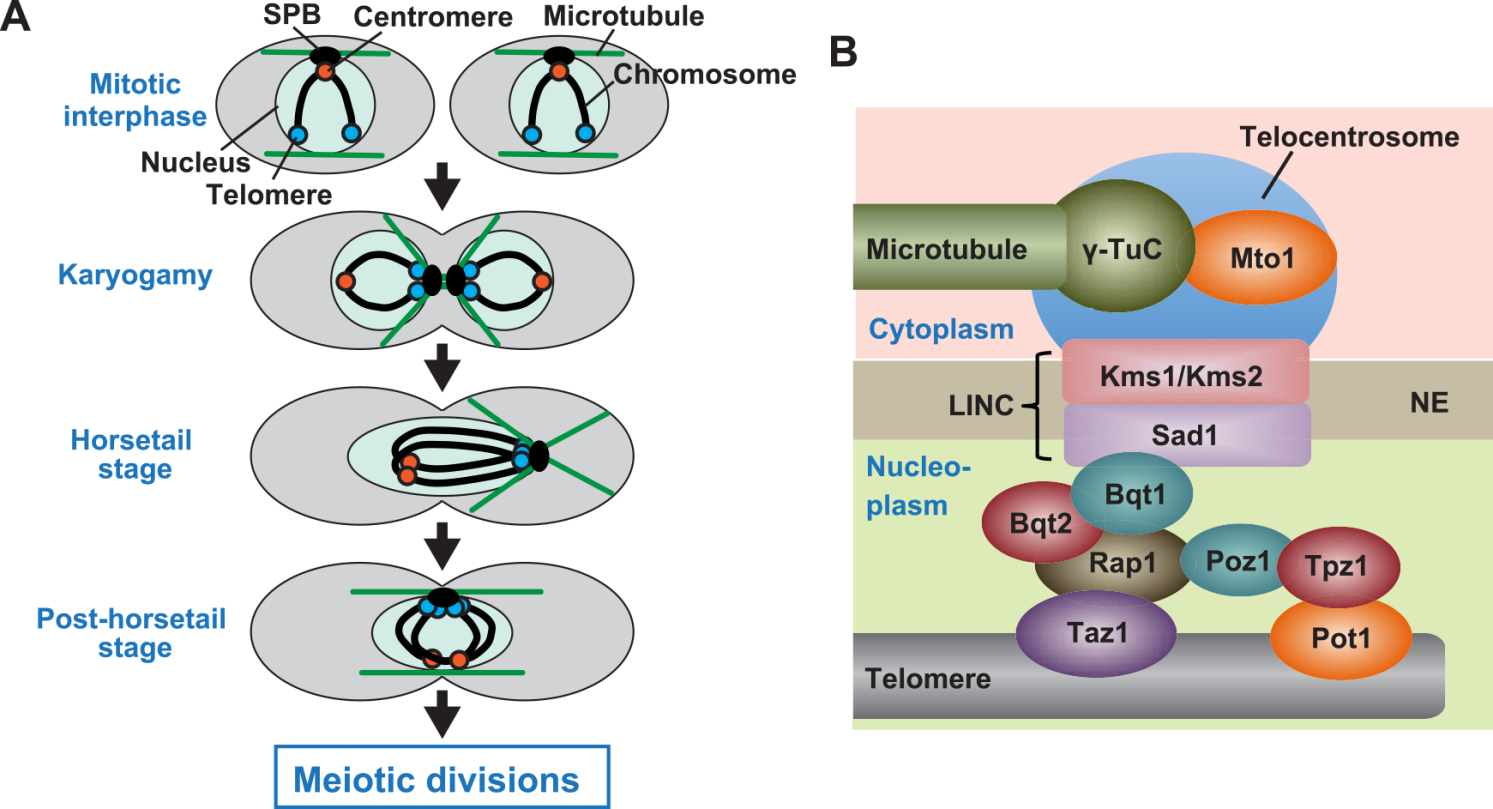
Changes in chromosome positioning during meiosis and LINC-dependent telocentrosome formation. (A) Changes in chromosome positioning during meiosis. During mitotic interphase, centromeres (red circles) are located at the SPB, while telomeres (blue circles) are located away from it. When cells enter meiosis by cell conjugation, telomeres cluster at the SPB, which radiates microtubules, and centromeres become detached from it (Karyogamy). After nuclear fusion, the diploid nucleus becomes elongated and oscillates between the cell ends, led by the SPB (Horsetail stage). Thereafter, the nucleus remains in the middle of the cell with several microtubules extending in parallel to the long cell axis until meiotic divisions start (Post-horsetail stage). (B) Factors required for telomere-LINC interaction and telocentrosome formation. γ-TuC: γ-tubulin complex; NE: nuclear envelope.

Among different organisms, the fission yeast *Schizosaccharomyces pombe* shows the most prominent example of the bouquet arrangement. *S*. *pombe* mitotic chromosomes are positioned with their centromeres clustered at the spindle pole body (SPB; a centrosome equivalent in fungi) and their telomeres located away from it (this corresponds to the “Rabl” configuration seen in other organisms) [31]. Under nitrogen-starved conditions, *S*. *pombe* cells enter meiosis through cell conjugation. Around this period, telomeres cluster at the SPB and centromeres become detached from it, forming the bouquet arrangement (Fig 1A) [32]. When the bouquet arrangement is formed, the SPB oscillates between the cell ends with the clustered telomeres, generating so-called “horsetail” nuclear movements (Fig 1A, Horsetail stage). The SPB-led telomere movements promote pairing of homologous chromosomes by inducing their alignment and contact; impairment of either telomere clustering or horsetail movements compromises homologous chromosome pairing [8, 33].

Studies of *S*. *pombe* have unveiled additional functions of the bouquet arrangement. Telomere clustering additionally contributes to spindle formation, and defective telomere clustering causes impairment of spindle formation [34, 35]. Furthermore, centromere detachment from the SPB induces the formation of meiosis-specific centromeres. During homologous chromosome segregation, homologous chromosomes attach to opposite SPBs (bipolar attachment) with sister chromatids attaching to the same SPB (monopolar attachment) (S1A Fig) [36, 37]. At this period, the kinetochores on the sister centromeres face the same side (kinetochore mono-orientation), facilitating monopolar attachment of sister chromatids, while centromere cohesion persists, preventing sister chromatid separation upon their bipolar attachment (S1B Fig) [38]. Persistent centromere cohesion also enables sister chromatid segregation (equational segregation) at meiosis II. Without centromere detachment from the SPB, meiotic centromere properties are not properly established and sister chromatids frequently undergo equational segregation at meiosis I [39, 40]. Centromere detachment also induces meiotic centromere formation in the budding yeast *Saccharomyces cerevisiae* [41].

The linker of nucleoskeleton and cytoskeleton (LINC) complex formed by the conserved SUN (Sad1/Unc-84) and KASH (Klarsicht/ANC-1/Syne homology) domain nuclear membrane proteins recently emerged as a key player in SPB association of telomeres and centromeres [8, 33, 42–44]. In *S*. *pombe*, the LINC complex consisting of Sad1 (SUN) and Kms1/Kms2 (KASH) is localized at the SPB. Upon entering meiosis, meiosis-specific Bqt1 and Bqt2 recruit the LINC complex to telomeres, resulting in localization of Sad1 and Kms1/Kms2 at telomeres in addition to the SPB [10, 45]. The telomere-recruited LINC complexes form the microtubule-organizing center termed the “telocentrosome” (Fig 1B). Subsequently, microtubule motors gather telomeres at the SPB by moving on SPB- and telocentrosome-nucleated microtubules (S1C Fig) [33, 45]. SPB localization of centromeres also depends on the LINC complex; the Csi1-dependent interaction of the outer kinetochore components with Sad1 causes tethering of mitotic centromeres to the SPB [44]. When the bouquet is formed, the outer kinetochore components delocalize from the centromere, and this kinetochore disassembly causes centromere detachment from the SPB [39, 46].

In *S*. *pombe*, centromere detachment immediately follows telomere clustering [47, 48], and both events require mating pheromone-dependent activation of MAP kinase [39, 49]. Together with the fact that SPB localization of telomeres and centromeres depends on the LINC complex, these facts suggest that regulation of centromere detachment and telomere clustering is related. However, their regulatory relationship remains unclear.

Here, we show that, when telomere clustering is inhibited, centromere detachment is also inhibited. Efficient centromere detachment requires SPB recruitment of the telomere component Taz1 by telomere clustering and microtubules that drive telomere clustering. We provide evidence indicating that this regulation secures proper spindle formation when telomere clustering is defective. To our knowledge, this is the first example of a regulatory relationship between telomere and centromere positions, which is critical for proper execution of meiotic divisions.

## Results

### Centromere detachment from the SPB is inhibited in telomere clustering-defective mutants

*S*. *pombe* cells normally enter meiosis through conjugation of two haploid cells. The bouquet arrangement is formed around the period of cell conjugation [45, 47], and persists until meiotic divisions start (Fig 1A) [32]. To explore the relationship between telomere clustering and centromere detachment in bouquet formation, we examined telomere and centromere positioning during karyogamy and the horsetail stage in telomere clustering-defective cells.

**Fig 2:**
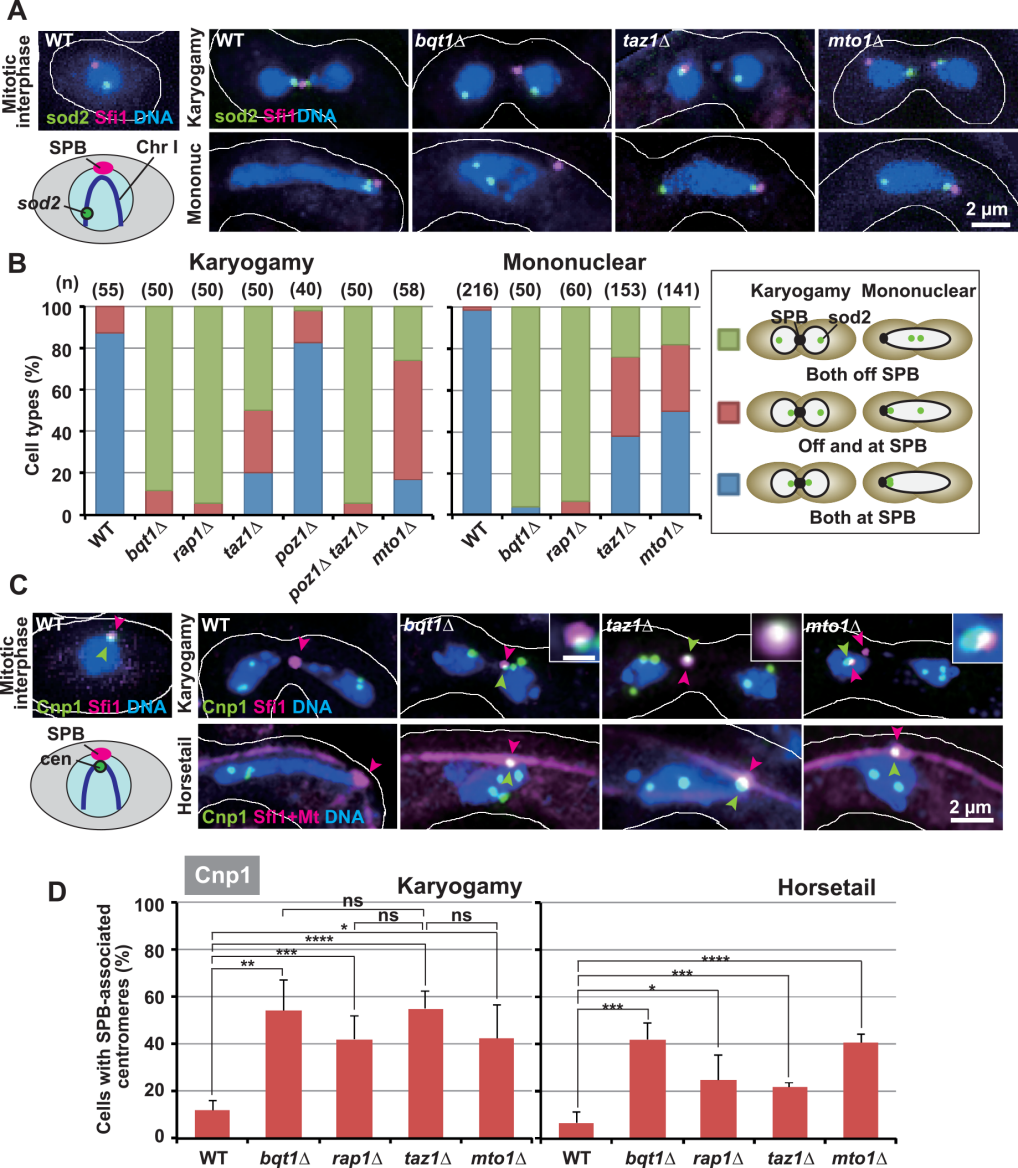
Telomere and centromere positioning in telomere clustering-defective cells. (A) Localization patterns of the telomere-adjacent *sod2* locus. The karyogamy stage was judged by two nuclei with teardrop or deformed shapes and/or with a single SPB (Karyogamy). The mononuclear stage, including both horsetail and post-horsetail stages, was judged by a single nucleus with a non-round, deformed shape (Mononuc). (B) Observation frequencies of different *sod2* localization patterns. Numbers in parentheses indicate the number of examined cells. (C) Centromere localization during karyogamy and the horsetail stage. Magenta and green arrowheads indicate the SPBs and SPB-co-localized centromeres, respectively. Insets are enlarged images of SPB-associated centromere signals (white bar: 0.5 μm). The karyogamy stage was judged as in (A). The horsetail stage was judged by a single nucleus with an astral microtubule array (Horsetail). (D) Population of cells containing SPB-associated centromeres. Averages of three independent experiments are shown. Error bars indicate standard deviation. More than 30 and 60 cells were examined in each experiment for karyogamy and the horsetail stage, respectively. Lines indicate sets of data that were statistically compared. *p*<*0.05; **p*<*0.01; ***p*<*0.005; ****p<0.001 (by the Student’s t-test); ns: no significant difference.

We first examined telomere positioning by visualizing the telomere-proximal *sod2* locus on chromosome I using the lacI/lacO recognition system [50] (Fig 2A, Mitotic interphase). In wild-type cells, during mitotic interphase, *sod2* signals were located away from the SPB, which was visualized by an mCherry-tagged, conserved SPB component, Sfi1 [51]. By contrast, they were mostly juxtaposed with the SPB during karyogamy and the meiotic mononuclear stage (including the horsetail and post-horsetail stages; see Fig 1A), confirming the occurrence of telomere clustering (Fig 2A and 2B, WT). Telomere clustering depends on telomere-LINC interaction, and a Sad1 interactor, Bqt1, and a telomere-Bqt1 connector, Rap1, are essential for this interaction (Fig 1B) [10, 11, 52]. Consistently, loss of either Bqt1 (*bqt1Δ*) or Rap1 (*rap1Δ*) caused almost complete elimination of *sod2*-SPB juxtaposition (Fig 2A and 2B). Furthermore, Rap1 interacts with telomeres via two different telomere-binding proteins, Taz1 and Pot1 (Fig 1B) [11, 52–54]. Loss of either Taz1 (*taz1Δ*) or a Rap1-Pot1 connector, Poz1 (*poz1Δ*), probably partially compromises telomere-LINC interaction, and loss of both eliminates the interaction completely. Accordingly, introduction of *taz1Δ* or *poz1Δ* mutation only reduced or rarely affected *sod2*-SPB juxtaposition, whereas introduction of both mutations almost completely eliminated their juxtaposition (Fig 2B, Karyogamy). Thus, telomere-clustering defects are correlated with telomere-LINC interaction defects. Telomere clustering also depends on telocentrosome formation, which requires a γ-tubulin complex(γ-TuC)-recruiting factor, Mto1 (Fig 1B) [45], and *sod2*-SPB juxtaposition was reduced in *mto1Δ* cells (Fig 2A and 2B).

We next examined centromere positioning by visualizing the centromere-specific histone H3 variant Cnp1 [55]. In wild-type cells, all centromeres were co-localized with the SPB during mitosis (Fig 2C, Mitotic interphase), but they were mostly located away from it during karyogamy and the horsetail stage (judged by a single nucleus with an astral microtubule array; see Fig 1A) (Fig 2C and 2D, WT). By striking contrast, in all telomere clustering-defective cells, centromeres were often co-localized with the SPB (Fig 2C and 2D; S2 Fig). Therefore, centromere detachment from the SPB is commonly inhibited in telomere clustering-defective cells. However, in *taz1Δ* and *mto1Δ* cells, although there was a considerable level of telomere-SPB association (Fig 2B), centromere detachment was inhibited at similar levels to those seen in *bqt1Δ* and *rap1Δ* cells during karyogamy (Fig 2D, Karyogamy; no statistically significant differences among the mutants), in which telomere-SPB association was almost completely lost (Fig 2B). This indicates that inhibition of centromere detachment is not correlated with loss of telomere-SPB association.

### Kinetochore disassembly is inhibited in telomere clustering-defective mutants

**Fig 3:**
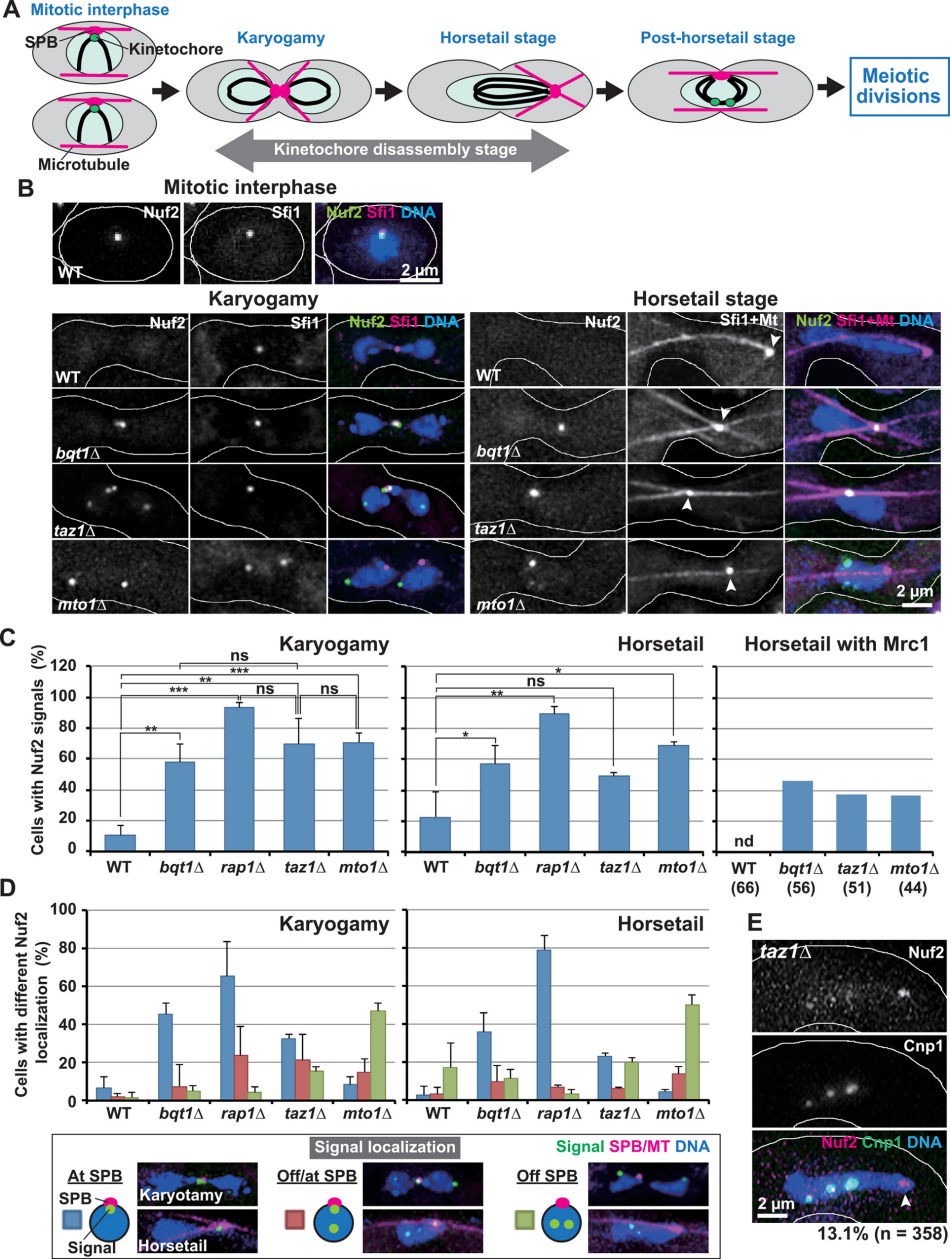
Localization of Nuf2 in telomere clustering-defective cells. (A) Kinetochore disassembly and meiosis progression. Green dots indicate intact kinetochores. The intact kinetochores are absent during almost the entire period of karyogamy and the horsetail stage (Kinetochore disassembly stage). (B) Nuf2 localization. The karyogamy and horsetail stages were judged, as in Fig 2C. Arrowheads in the horsetail stage show SPB positions. (C) Population of cells containing Nuf2 signals. Karyogamy: karyogamy stage; Horsetail: the horsetail stage; Horsetail with Mrc1: the horsetail stage with nuclear Mrc1 signals. Numbers in parentheses show the number of examined cells. Bars show averages of three independent experiments except for those in “Horsetail with Mrc1”. More than 30 and 60 cells were examined in each experiment for karyogamy and the horsetail stage, respectively. Error bars indicate standard deviation. Lines indicate sets of data that were statistically compared. *p*<*0.05; **p*<*0.005; ***p*<*0.0005; ns: no significant difference (by the Student’s t-test); nd: not detected. (D) Population of cells with different Nuf2 localization. At SPB: one SPB-associated signal; Off/at SPB: SPB-associated and SPB-dissociated signals; Off SPB: SPB-dissociated signals. Images in the box show the representative localization of the SPB and signals. (E) A Nuf2 signal dissociated from the centromere in the *taz1Δ* mutant (arrowhead). The percentage indicates the observation frequency of centromere-dissociated Nuf2 signals.

Because centromere detachment is induced by outer kinetochore disassembly, we next examined the localization of Nuf2 and Mis12, which are components of the Ndc80 and Mis12 outer kinetochore complexes, respectively. The outer kinetochore components delocalize from the centromere at the time of centromere detachment, and remain delocalized until kinetochore reformation occurs around the end of the horsetail stage (Fig 3A) [46]. In wild-type cells, Nuf2 was observed at the centromere-localized SPB during mitotic interphase, and was mostly absent during karyogamy and the horsetail stage (Fig 3B and 3C, WT). Mis12 showed similar localization patterns, but with higher observation frequencies (S3A and S3B Fig, WT). The higher observation frequencies probably mean that Mis12 complexes delocalize from the centromere later and re-localize at the centromere earlier than Ndc80 complexes.

By contrast, in all telomere clustering-defective mutants, Nuf2 and Mis12 signals were frequently observed (Fig 3B–3D; S3 Fig). The higher observation frequencies in the horsetail stage were not due to enrichment of cells in the late, kinetochore-reformation stage (Fig 3A) because, when we specified early horsetail-stage cells by nuclear signals of RFP-tagged Mrc1, a DNA replication regulator that was observed only in the early meiotic stage (S4A Fig), Nuf2 was still observed at higher frequencies (Fig 3C, Horsetail with Mrc1). In addition, the higher observation frequencies were not caused by centromere clustering-dependent increases in signal intensities because the number of centromere signals significantly increased during the horsetail stage in the *bqt1Δ* mutant [average number of Cnp1 signals was 3.4 ± 1.1 (n = 162) for wild type and 3.9 ± 1.2 (n = 225) for the *bqt1Δ* mutant during the horsetail stage; p<0.001 by the Student’s t-test]. This indicates that delocalization of the outer kinetochore components is inhibited in the mutants. The retained signals were localized either at the SPB or at the SPB-detached centromeres (Fig 3B and 3D; S3A, S3C and S4B Figs) [centromere and/or SPB localization of Nuf2 signals was confirmed by co-localization of the Nuf2 signals with either SPB and/or centromere signals in cells in which both the SPB and centromeres were visualized (n = 73)]. Furthermore, the signals were sometimes co-localized with the centromere-free SPB (Fig 3E, arrowhead). This indicates that, in addition to the dissociation of kinetochore complexes from the centromere, that from the SPB is also inhibited. We also noticed that localization patterns of the retained signals in the *mto1Δ* mutant were somewhat different from those in other mutants; the signals tended to be localized at SPB-detached centromeres in *mto1Δ* cells, but at the SPB in other mutant cells (Fig 3D; S3C and S4B Figs).

### Microtubule disruption inhibits centromere detachment from the SPB

Because telomere clustering depends on microtubules [45], we next examined if microtubule disruption inhibits centromere detachment using a microtubule-depolymerizing drug, methyl 2-benzimidazole carbamate (MBC). To disrupt microtubules before telomere clustering, we synchronously induced meiosis using haploid cells bearing both mating-type genes, as reported previously [40, 45]. These haploid cells underwent meiosis fairly synchronously after a single mitotic division (shown by a transient increase in binuclear cells) in nitrogen-depleted conditions, forming more than two nuclei (S5A–S5C Fig). We treated the cells with MBC or its solvent DMSO after mitotic division.

**Fig 4:**
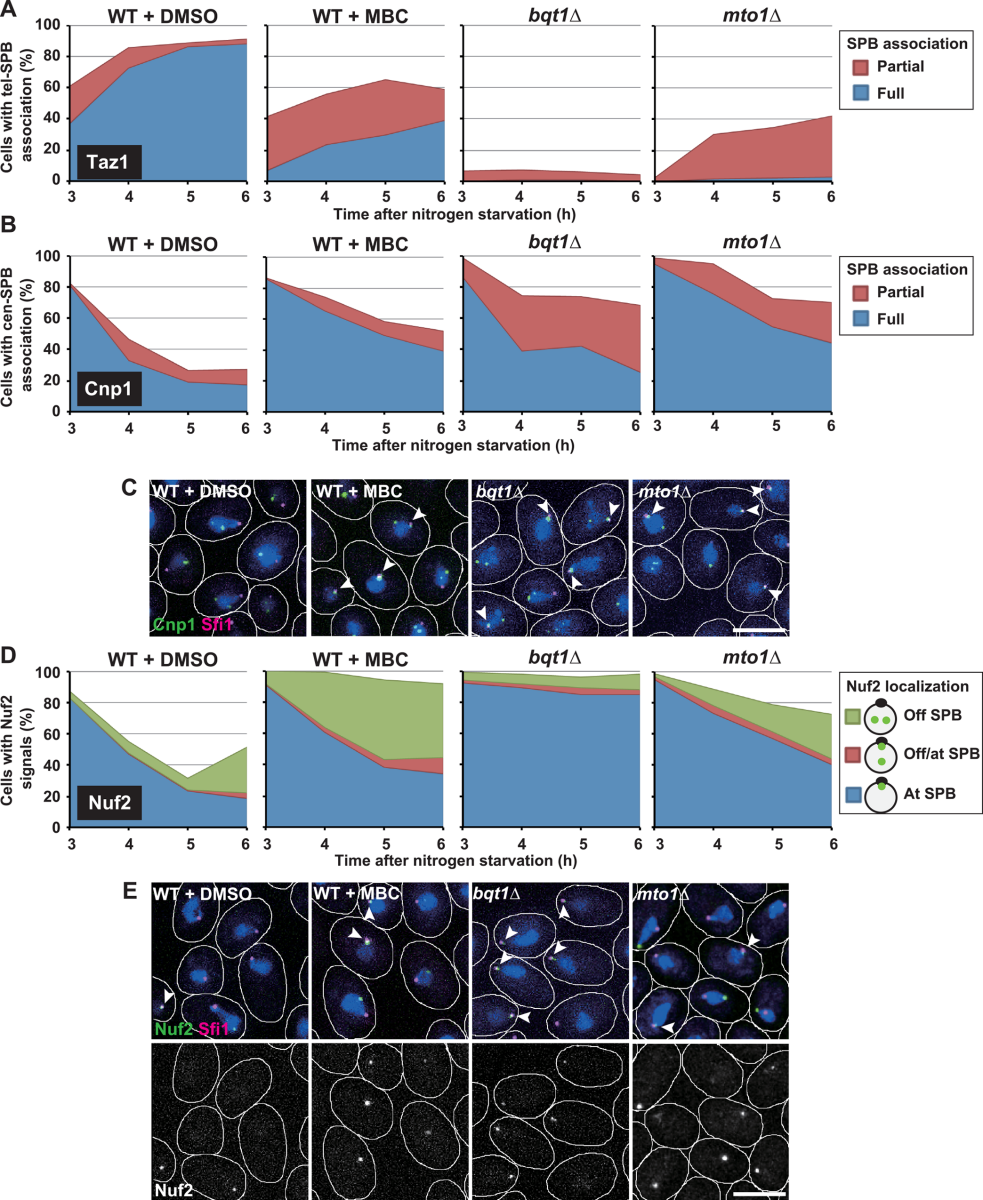
Effects of MBC on centromere detachment and kinetochore disassembly. (A and B) Changes in the population of haploid cells with SPB-associated telomeres (A) or centromeres (B) during meiosis progression. SPB association of a portion of (Partial) or all (Full) Taz1/Cnp1 signals is shown by red or blue, respectively. (C) Centromere positioning in haploid cells. (D) Changes in the population of haploid cells containing Nuf2 signals during meiosis progression. Nuf2 localization is shown as in Fig 3D. (E) Nuf2 localization in haploid cells. In (C) and (E), images show cells incubated in nitrogen-free medium for 5 h. White lines show cell outlines, and arrowheads show Cnp1 or Nuf2 signals co-localized with SPB signals. Bars: 5 μm. WT+DMSO: wild-type haploid cells treated with DMSO; WT+MBC: wild-type haploid cells treated with MBC.

In the DMSO-treated control experiment, as the percentage of cells with SPB-associated telomeres increased (Fig 4A, WT+DMSO), the percentage of those with SPB-associated centromeres (Fig 4B and 4C, WT+DMSO) or Nuf2 signals (Fig 4D and 4E, WT+DMSO) conversely decreased. This confirms the occurrence of centromere detachment and kinetochore disassembly in addition to telomere clustering. It should also be noted that loss of Bqt1 or Mto1 caused inhibition of telomere clustering, centromere detachment, and kinetochore disassembly in haploid meiotic cells, as shown by the decrease in cells with SPB-associated telomeres (Fig 4A, *bqt1Δ* and *mto1Δ*) and the increase in those with SPB-associated centromeres (Fig 4B and 4C, *bqt1Δ* and *mto1Δ*) or Nuf2 signals (Fig 4D and 4E, *bqt1Δ* and *mto1Δ*). Thus, haploid meiotic cells mirror diploid meiotic zygotes.

MBC treatment inhibited telomere clustering without inhibiting meiosis progression (S5A–S5C Fig), as shown by a significant decrease in the percentage of cells with SPB-associated telomeres (Fig 4A, WT+MBC). Notably, MBC also inhibited centromere detachment and kinetochore disassembly, as shown by a significant increase in the percentage of cells with SPB-associated centromeres (Fig 4B and 4C, WT+MBC) or Nuf2 signals (Fig 4D and 4E, WT+MBC). The retained Nuf2 signals were often located away from the SPB (Fig 4D and 4E, WT+MBC). This localization pattern was similar to that seen in *mto1Δ* cells but different from that seen in *bqt1Δ* cells, where the signals were mainly at the SPB (Fig 4D and 4E). These observations suggest that the effects of MBC on kinetochore disassembly are similar to those of *mto1Δ* mutation but different from those of *bqt1Δ* mutation. Furthermore, although the effects are similar, those of MBC are perhaps stronger than the *mto1Δ* mutation because the percentage of Nuf2-retaining cells was larger in MBC-treated cells than in *mto1Δ* cells (Fig 4D, WT+MBC and *mto1Δ*).

### Nuf2 delocalization is delayed and its re-localization is advanced in telomere clustering-defective cells

**Fig 5:**
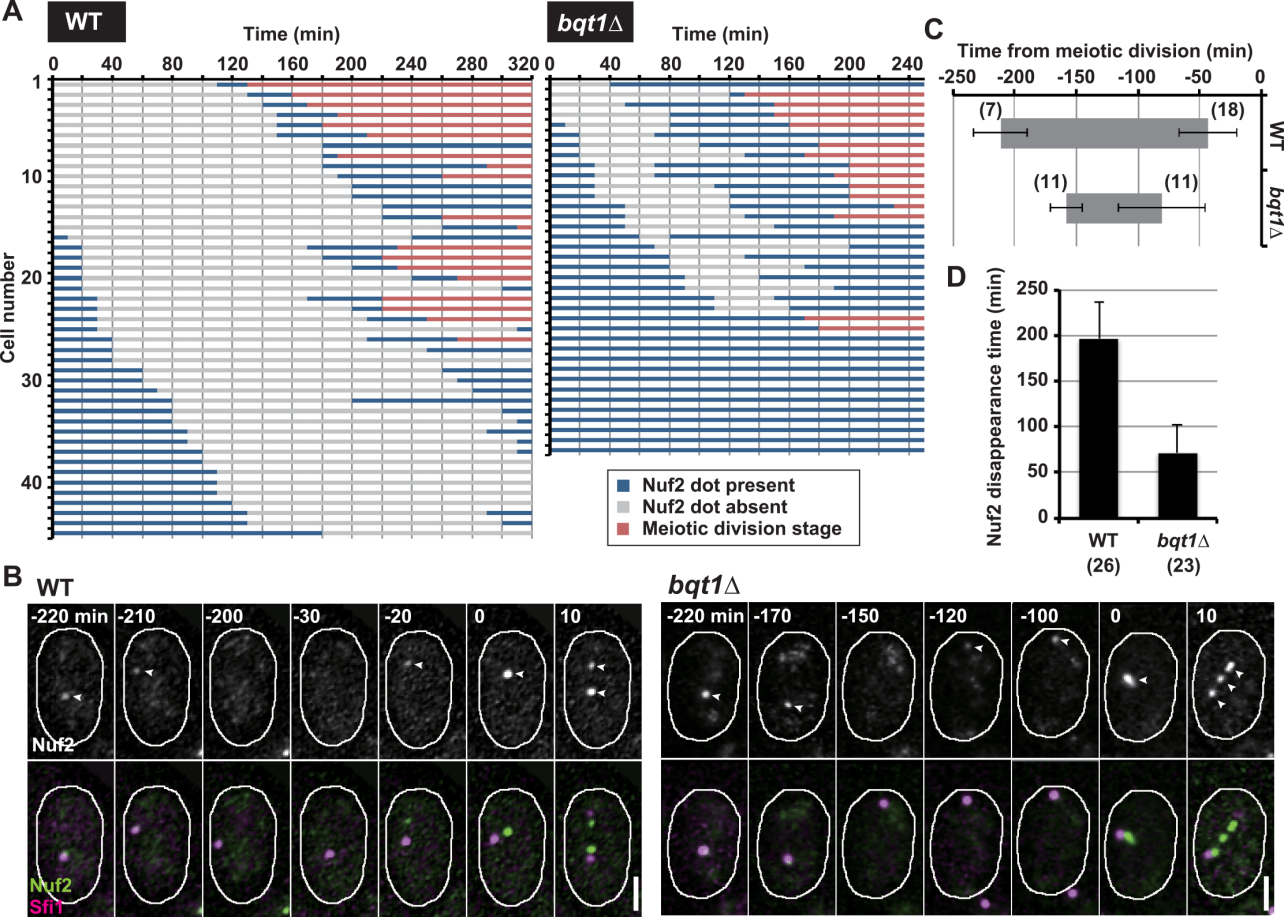
Live cell analysis of Nuf2 localization in haploid meiotic cells. (A) Live cell analysis of Nuf2 localization in haploid wild-type and *bqt1Δ* cells. Cells were induced to enter meiosis by incubation in nitrogen-free medium at 30°C. After incubation for 4 h, they were observed every 10 min at 25°C. Each bar indicates Nuf2 localization in each meiotic cell. Blue and gray bars indicate periods when Nuf2 dots were present and absent, respectively, and red bars indicate the meiotic division stage. (B) Changes in Nuf2 localization in haploid wild-type and *bqt1Δ* cells during meiosis. Numbers show time from the start of meiosis I, and arrowheads show Nuf2 signals. White bars: 2 μm. (C) Nuf2 disappearance period relative to the start of meiosis I in haploid cells (grey bars). (D) Duration of Nuf2 disappearance in haploid meiosis. Numbers in parentheses and error bars indicate the number of examined cells and standard deviation, respectively.

To understand the effects of impaired telomere clustering on kinetochore disassembly in greater detail, we next examined the localization dynamics of kinetochore components in haploid meiotic cells. In all wild-type cells, Nuf2 signals disappeared transiently (Fig 5A and 5B, WT; S1 and S2 Movies). By contrast, in *bqt1Δ* cells, Nuf2 signals sometimes did not disappear (Fig 5A, *bqt1Δ*), confirming inhibition of kinetochore disassembly. Furthermore, even when they disappeared, their disappearance was delayed, and their reappearance was conversely advanced (Fig 5A–5C; S3 and S4 Movies); as a result, the duration of Nuf2 disappearance was shorter (Fig 5D). These observations confirm the inhibition of kinetochore disassembly in telomere clustering-defective cells.

### Defective telomere clustering causes equational segregation of sister chromatids at meiosis I

Kinetochore disassembly is thought to be required for establishment of sister kinetochore mono-orientation and protection of centromere cohesion; therefore, we next examined sister chromatid segregation in telomere clustering-defective cells. For this analysis, we used cells that do not form chiasmata because chiasmata promote monopolar attachment of sister chromatids and obscure centromere properties (S1B Fig) [38]. If the properties of meiosis-specific centromeres are impaired, sister chromatids should undergo equational segregation in chiasma-lacking cells.

**Fig 6:**
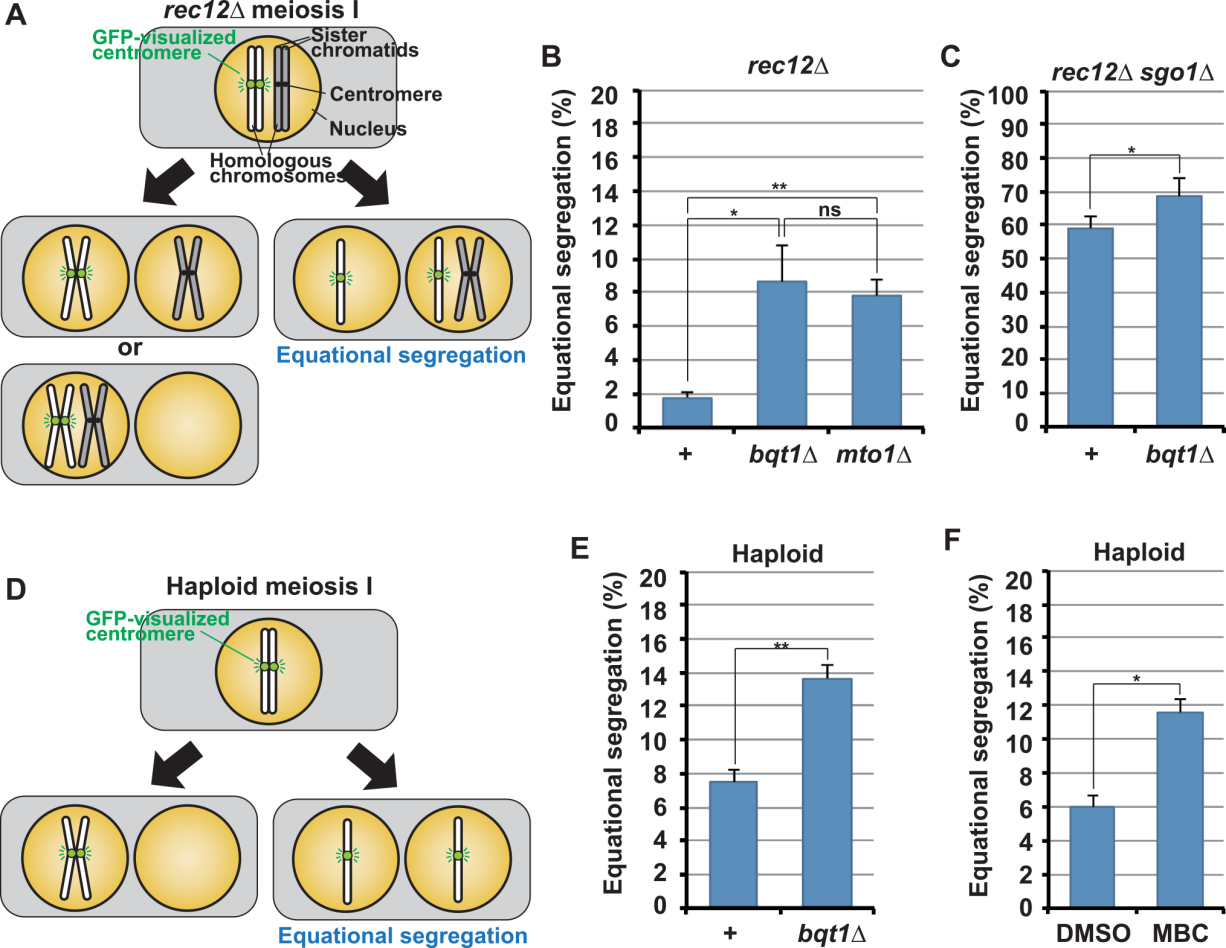
Sister chromatid segregation in telomere clustering-defective cells. (A) Chromosome segregation patterns at meiosis I in *rec12Δ* diploid zygotes. (B and C) The frequencies of equational segregation of sister chromatids at meiosis I in *rec12Δ* (B) and *rec12Δ sgo1Δ* (C) diploid zygotes. (D) Chromosome segregation patterns at meiosis I in haploid cells. (E and F) Effects of *bqt1Δ* mutation (E) or MBC treatment (F) on sister chromatid segregation in haploid meiotic cells. Sister chromatid segregation was analyzed by visualizing the centromere-proximal region of chromosome II [40]. +: no mutations otherwise depicted; DMSO: treated with DMSO; MBC: treated with MBC. Averages of three independent experiments are shown. Error bars indicate standard deviation. More than 60 cells were examined in each experiment. Lines indicate pairs of data that were statistically compared. *p<0.05; **p<0.005; ns: no significant difference (by the Student’s t-test).

GFP visualization of centromeres of one of the homologous chromosomes (Fig 6A) showed that sister chromatids rarely underwent equational segregation in *rec12* recombination-deficient, chiasma-lacking zygotes (Fig 6B, +), as reported previously [38, 40]. By contrast, introduction of *bqt1Δ* or *mto1Δ* mutation significantly increased equational segregation in *rec12* zygotes (Fig 6B). This indicates that the centromere properties are compromised. In chiasma-lacking cells, sister centromeres frequently attach to both SPBs despite mono-orientation of sister kinetochores, but protected centromere cohesion prevents sister chromatid separation. In these cells, *sgo1Δ* mutation, which eliminates cohesion protection, causes frequent equational segregation of sister chromatids (Fig 6C, +) [38]. Notably, *bqt1Δ* mutation further increased equational segregation in the *rec12Δ sgo1Δ* mutant (Fig 6C, *bqt1Δ*), similar to *moa1Δ* mutation (S6 Fig), which is thought to compromise kinetochore mono-orientation [56]. This strongly suggests that *bqt1Δ* mutation compromises kinetochore mono-orientation. *bqt1Δ* mutation also increased equational segregation in haploid *rec12*^*+*^ cells (Fig 6D and 6E), indicating that the increase was not attributed to loss of Rec12 functions. Furthermore, MBC treatment also increased equational segregation (Fig 6F). All these results support the idea that impairment of telomere clustering causes inhibition of kinetochore disassembly that is required for establishment of the meiosis-specific centromere properties.

### SPB-recruited Taz1 promotes centromere detachment from the SPB

**Fig 7:**
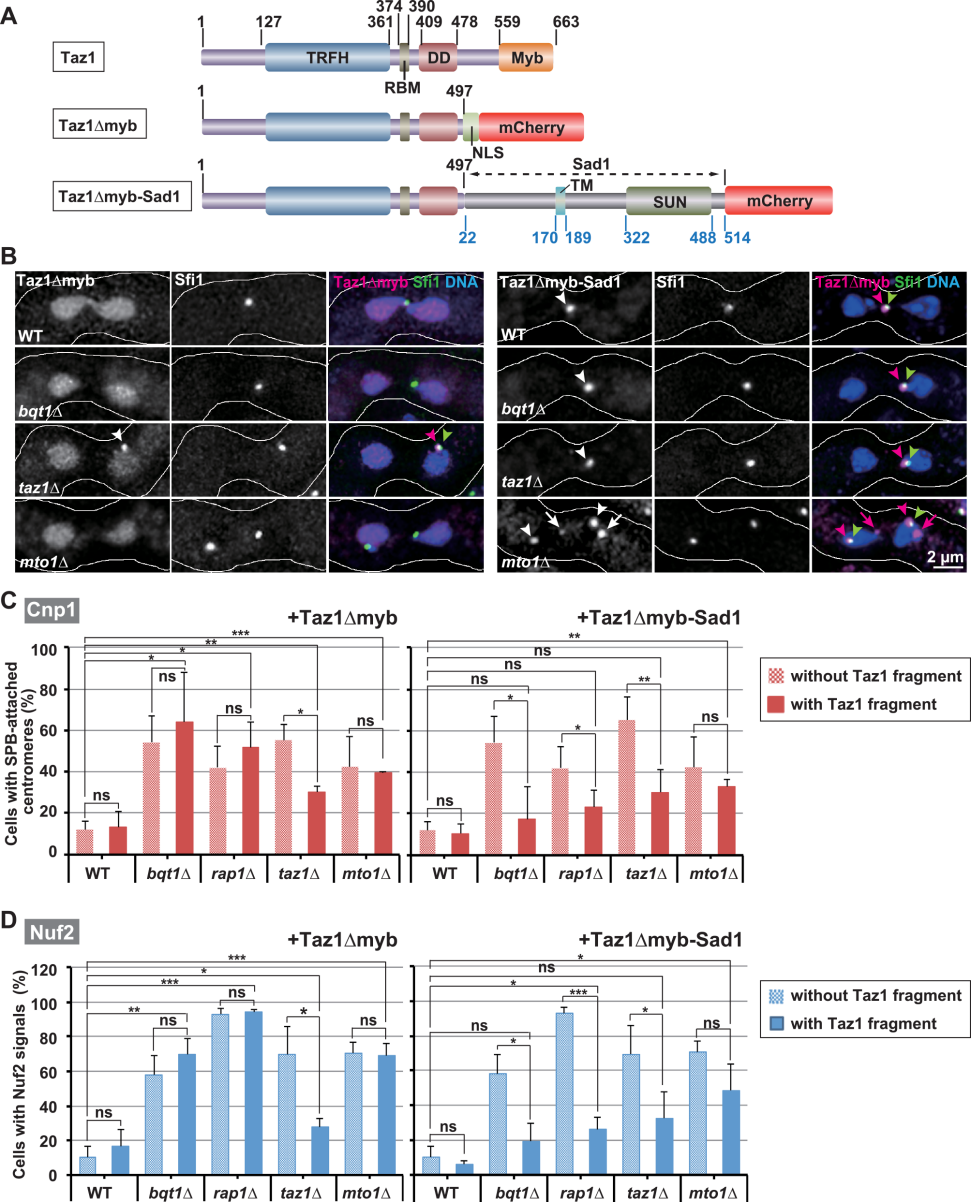
Effects of a Taz1 fragment on centromere positioning and kinetochore disassembly. (A) Schematic diagrams of Taz1, Taz1*Δ*myb, and Taz1*Δ*myb-Sad1. Black and blue numbers indicate amino acid numbers of Taz1 and Sad1, respectively. TRFH: TRF homology domain; RBM: Rap1-binding motif; DD: dimerization domain; Myb; Myb DNA-binding domain; NLS: nuclear localization sequence; TM: transmembrane domain; SUN: SUN domain [57] (http://www.pombase.org/spombe/result/SPBC12D12.01). Note that Sad1 lacks its N-terminal 21 amino acids. (B) Intracellular localization of Taz1*Δ*myb (left) and Taz1*Δ*myb-Sad1 (right) during karyogamy. Arrowheads show co-localization of SPB and Taz1*Δ*myb/Taz1*Δ*myb-Sad1 signals, and arrows show SPB-dissociated Taz1*Δ*myb-Sad1 signals. (C and D) Population of cells containing SPB-associated centromeres (C) or Nuf2 signals (D) during karyogamy. Averages of three independent experiments are shown. At least 30 cells were examined in each experiment. Error bars: standard deviation. Shaded bars indicate the data adopted from Figs 2D and 3C. Lines indicate sets of data that were statistically compared. *p<0.05, **p*<*0.005, ***p<0.0005; ns: no significant difference (p>0.05) (Student’s t-test). In all experiments, the fusion gene of Taz1*Δ*myb or Taz1*Δ*myb-Sad1 was integrated at the *aur1*^*+*^ locus on the chromosome and expressed under the *taz1*^*+*^ promoter.

Telomere clustering causes recruitment of telomere-LINC connectors to the SPB (S7A Fig, Wild type). We therefore hypothesized that an SPB-recruited telomere-LINC connector(s) promotes centromere detachment. Among the telomere-LINC connectors, we suspected that Taz1 contributes to centromere detachment, because in the *taz1Δ* mutant, although telomere-SPB association was substantially retained (Fig 2B, *taz1Δ*), the observed inhibition of centromere detachment was not statistically different from that observed in *bqt1Δ* or *rap1Δ* cells during karyogamy (Figs 2D and 3C, S3B Fig), where telomere-SPB association was almost completely eliminated. To test this possibility, we constructed a Taz1 fragment that lacks the telomeric DNA-binding domain (Myb domain) [11, 57] and fused it with an mCherry fluorescent protein for its visualization and with the nuclear localization sequence for its nuclear localization (Taz1*Δ*myb; Fig 7A). In *taz1Δ* cells, Taz1*Δ*myb was expected to be localized at the SPB by interacting with SPB-localized Rap1 [11], but not with telomeres (S7A Fig, *taz1Δ+*Taz1*Δ*myb). If SPB recruitment of Taz1 is important, it should restore centromere detachment in *taz1Δ* cells.

In wild-type cells, Taz1*Δ*myb was localized in the nucleus during karyogamy with a very low or undetectable level of accumulation at telomeres or the SPB (Fig 7B, WT). Its weak SPB accumulation was probably due to the presence of endogenous intact Taz1. In *taz1Δ* cells, by contrast, Taz1*Δ*myb accumulated at the SPB (Fig 7B, *taz1Δ*, arrowheads) without restoring telomere-SPB association (S7B Fig, +Taz1*Δ*myb, *taz1Δ*). Importantly, Taz1*Δ*myb significantly decreased the percentage of cells with SPB-associated centromeres or Nuf2 signals (Fig 7C and 7D, +Taz1*Δ*myb, *taz1Δ*). This result supports the idea that defective SPB recruitment of Taz1 causes inhibition of centromere detachment in *taz1Δ* cells.

In *bqt1Δ* and *rap1Δ* cells, Taz1*Δ*myb failed to decrease cells with SPB-associated centromeres or Nuf2 signals (Fig 7C and 7D, +Taz1*Δ*myb). However, Taz1*Δ*myb did not accumulate at the SPB because Taz1 cannot directly interact with the LINC complex without Bqt1 or Rap1 [10] (Fig 7B, Taz1*Δ*myb, *bqt1Δ*), and it remained unclear if defective SPB recruitment of Taz1 is the cause of the inhibition in these cells. To elucidate this point, we artificially tethered Taz1*Δ*myb to the SPB by fusing it with almost the entire length of Sad1, a LINC component that constitutively localizes at the SPB (Taz1*Δ*myb-Sad1; Fig 7A; S7A Fig, *bqt1Δ+*Taz1*Δ*myb-Sad1). Taz1*Δ*myb-Sad1 did not affect cell growth (judged by cell viability and the doubling time) and centromere-SPB association during mitosis. Importantly, Taz1*Δ*myb-Sad1 accumulated at the SPB without restoring telomere-SPB association in *bqt1Δ* and *rap1Δ* cells, as well as in *taz1Δ* cells (Fig 7B and S7B Fig, *+*Taz1*Δ*myb-Sad1), and significantly decreased the percentage of cells with SPB-associated centromeres or Nuf2 signals (Fig 7C and 7D, +Taz1*Δ*myb-Sad1). These observations indicate that a lack of Taz1 at the SPB causes inhibition of centromere detachment and kinetochore disassembly in *bqt1Δ*, *rap1Δ*, and *taz1Δ* cells. Taking all these results together, we conclude that SPB-recruited Taz1 promotes centromere detachment from the SPB. It should be noted, however, that in *bqt1Δ*, *rap1Δ*, or *taz1Δ* cells expressing Taz1*Δ*myb-Sad1, the percentage of cells with SPB-associated centromeres or Nuf2 signals was slightly higher than that in wild-type cells (Fig 7C and 7D), suggesting that Taz1*Δ*myb-Sad1 does not completely restore centromere detachment.

### Microtubules promote centromere detachment from the SPB independently of SPB-recruited Taz1

**Fig 8:**
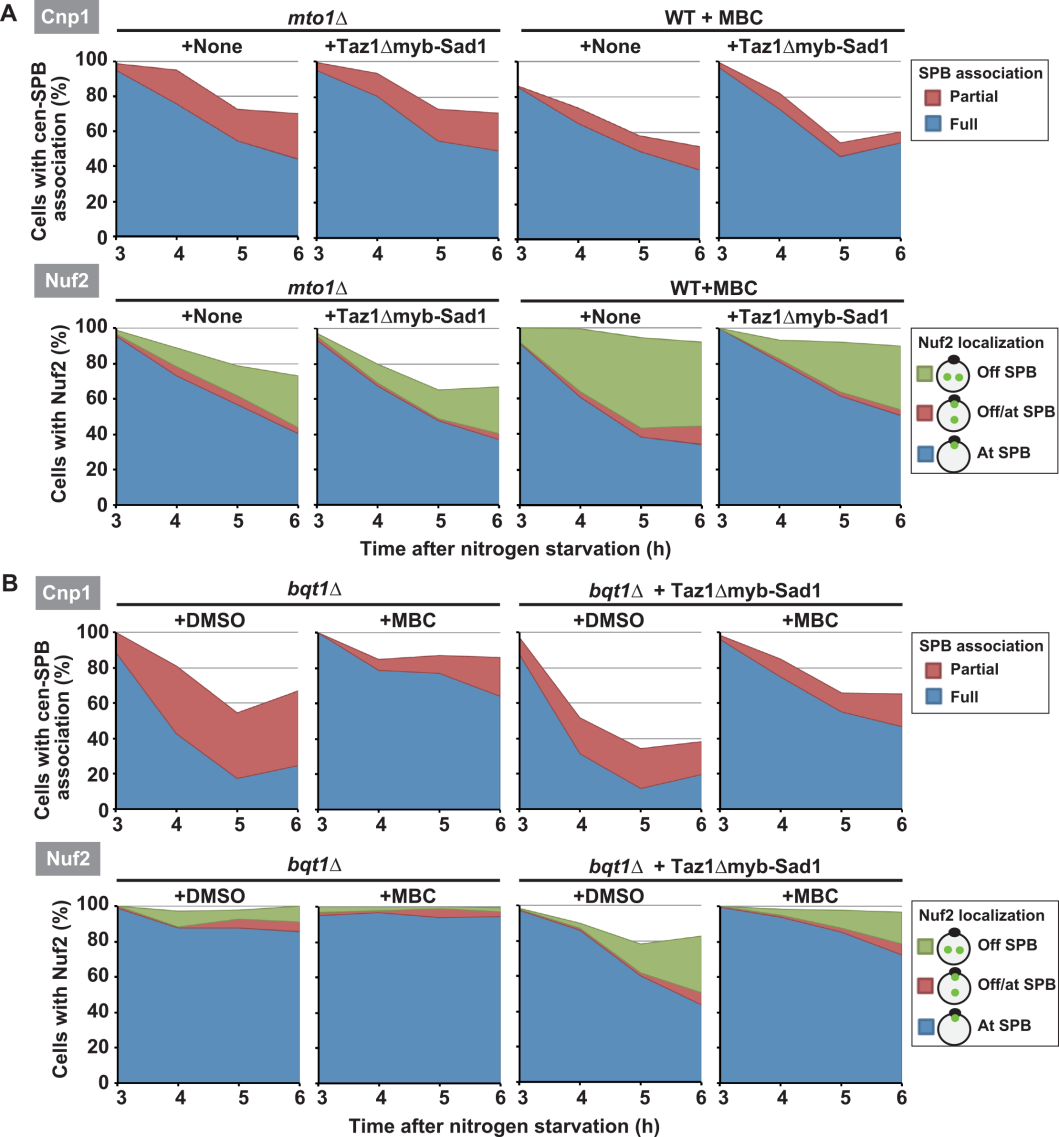
Taz1-independent inhibition of centromere detachment from the SPB and kinetochore disassembly by microtubule disruption. (A) Effects of Taz1Δmyb-Sad1 on centromere (Cnp1) and Nuf2 (Nuf2) localization in *mto1Δ* (*mto1Δ*) or MBC-treated wild-type (WT+MBC) cells. Data of *mto1Δ* or MBC-treated wild-type cells without Taz1Δmyb-Sad1 expression (+None) are adopted from Fig 4. +None: without Taz1Δmyb-Sad1 expression; +Taz1Δmyb-Sad1: with Taz1Δmyb-Sad1 expression. (B) Effects of MBC on centromere (Cnp1) and Nuf2 (Nuf2) localization in *bqt1Δ* cells with (*bqt1Δ+*Taz1Δmyb-Sad1) or without (*bqt1Δ*) Taz1Δmyb-Sad1 expression. +DMSO: treated with DMSO; +MBC: treated with MBC.

In *mto1Δ* or MBC-treated wild-type cells, although Taz1 is often recruited to the SPB by frequent telomere-SPB association (Figs 2B and 4A) [45], centromere detachment was substantially inhibited (Figs 2D and 4B). In addition, in *mto1Δ* zygotes, although Taz1*Δ*myb-Sad1 was localized at the SPB (Fig 7B, +Taz1*Δ*myb-Sad1, *mto1Δ*, arrowheads), it decreased the percentage of cells with SPB-associated centromeres or Nuf2 signals only slightly, and the decrease was not statistically significant (Fig 7C and 7D, +Taz1*Δ*myb-Sad1, *mto1Δ*). Likewise, in *mto1Δ* or MBC-treated haploid meiotic cells, Taz1*Δ*myb-Sad1 did not significantly affect centromere and Nuf2 localization (Fig 8A) as well as meiosis progression (S5D and S5E Fig, WT+Taz1*Δ*myb-Sad1 and *mto1Δ+*Taz1Δmyb-Sad1). These results suggest that microtubules contribute to the regulation of centromere detachment independently of Taz1.

To test this possibility, we treated haploid *bqt1Δ* cells, which are completely defective in SPB recruitment of Taz1, with MBC. MBC treatment increased the percentage of cells containing SPB-associated centromeres (Fig 8B, Cnp1, *Δbqt1*) without inhibiting meiosis progression (S5D Fig, *bqt1Δ*). Even with Taz1Δmyb-Sad1 expression, MBC treatment increased the percentage of cells with Nuf2 signals, as well as those with SPB-associated centromeres (Fig 8B, *bqt1Δ*+Taz1Δmyb-Sad1). From these results, we conclude that microtubules promote centromere detachment independently of SPB-recruited Taz1. Microtubules drive SPB movements, which cause horsetail nuclear movements. However, centromere detachment was not dependent on SPB movements because centromere detachment was not inhibited in *dhc1Δ* cells, which are defective in SPB movements [58] (S2 Fig).

### Induction of centromere detachment causes spindle impairment in telomere clustering-defective cells

Our results show the presence of regulatory mechanisms that inhibit centromere detachment from the SPB in the absence of telomere-SPB association. However, the advantages of the mechanisms in meiosis are unclear. It was recently reported that defective telomere clustering causes impairment of spindle formation with detachment of the SPB from the nuclear membrane, and that centromere-SPB association restores spindle formation in the absence of telomere clustering [34, 35]. These facts suggest that meiotic spindle formation requires SPB association of either telomeres or centromeres, and that concurrent detachment of telomeres and centromeres from the SPB is harmful for spindle formation. The inhibitory mechanisms of centromere detachment may secure spindle formation by preventing concurrent detachment of centromeres and telomeres. To test this possibility, we observed spindle formation in *taz1Δ* and *bqt1Δ* mutants, which are partially and fully defective in telomere clustering, respectively (Fig 2B), and examined the effects of Taz1Δmyb-Sad1 on spindle formation. If spindle formation requires SPB association of either telomeres or centromeres, Taz1Δmyb-Sad1-dependent elimination of centromere-SPB association should cause impairment of spindle formation in telomere clustering-defective cells, and the extents of the resultant impairments should be correlated with the extents of telomere-clustering defects.

**Fig 9:**
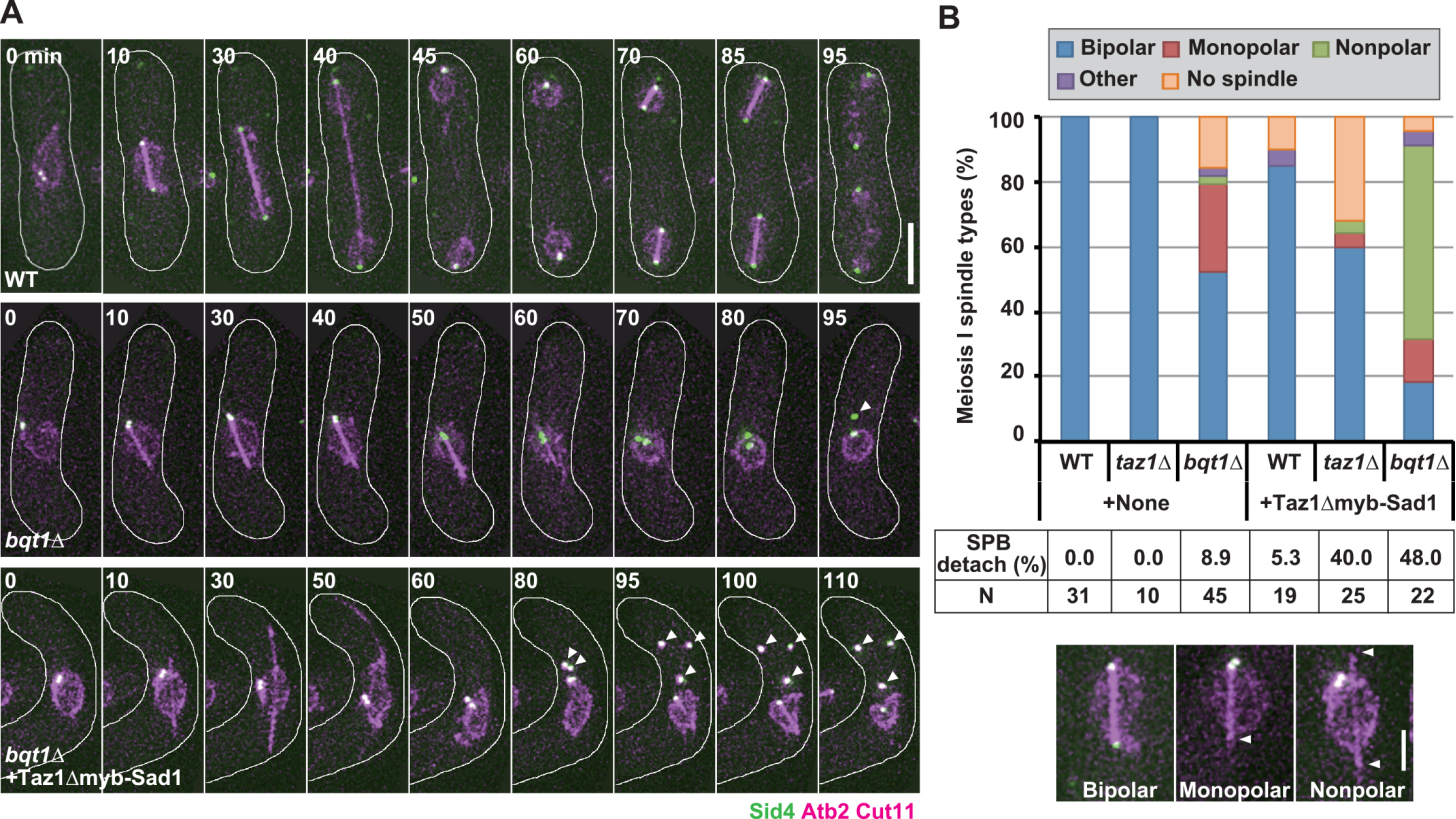
Effects of Taz1Δmyb-Sad1 on spindle formation in telomere clustering-defective cells. (A) Dynamics of meiotic spindles and the SPB. Microtubules, the SPB, and the nuclear periphery were visualized by mCherry-tagged α-tubulin (Atb2), a GFP-tagged SPB component Sid4, and an mCherry-tagged nuclear pore component Cut11 [59], respectively. Images were taken every 5 min. Arrowheads indicate SPBs that are dissociated from the nuclear periphery. White lines indicate cell outlines, and numbers indicate time in minutes. +Taz1Δmyb-Sad1: with Taz1Δmyb-Sad1 expression. Bar: 5 μm. (B) Observation frequencies of various meiosis-I spindle types and SPB detachment from the nuclear periphery. +None: without Taz1Δmyb-Sad1 expression; +Taz1Δmyb-Sad1: with Taz1Δmyb-Sad1 expression. Bipolar: a normal spindle with SPBs at both poles; Monopolar: a spindle with the SPB at one of the poles; Nonpolar: an SPB-lacking spindle; Other: other types of spindles; No spindle: no spindle formation; SPB detach: percentages of cells with the SPB detached from the nuclear periphery; N: number of examined cells. Typical images of the observed spindles are shown at the bottom, and arrowheads indicate the spindle pole lacking a SPB. Bar: 2 μm.

In wild-type cells, meiosis I and II occurred sequentially with the formation of one and two spindles, respectively (Fig 9A, WT; S5 Movie), and Taz1Δmyb-Sad1 only marginally compromised spindle formation (Fig 9B, WT). In *taz1Δ* cells, spindle impairment was not detected, while, in *bqt1Δ* cells, impaired spindle formation together with detachment of the SPB from the nuclear periphery were observed in about half of cases, as reported previously [34] (Fig 9A and 9B, +None; S6 Movie) (we cannot exclude the possibility that the detached SPBs remained connected with the nucleus by Cut11-lacking nuclear membrane). Notably, Taz1Δmyb-Sad1 induced or increased spindle impairment together with SPB detachment from the nuclear periphery in *taz1Δ* and *bqt1Δ* cells (Fig 9A and 9B, +Taz1Δmyb-Sad1; S7 Movie), supporting the importance of centromere-SPB association for spindle formation. In addition, the extents of spindle impairment were correlated with the extents of defective telomere clustering; telomere clustering was more severely impaired in *bqt1Δ* cells than in *taz1Δ* cells (Fig 9B, +Taz1Δmyb-Sad1). These results support the idea that meiotic spindle formation requires SPB association of either telomeres or centromeres, and that the inhibitory mechanisms of centromere detachment secure spindle formation by preventing the concurrent detachment of centromeres and telomeres. The SPB was frequently missing at one (monopolar) or both (nonpolar) spindle poles (Fig 9A and 9B; S6 and S7 Movies). This may mean that telomere/centromere-SPB association is critical for anchoring the SPB to the spindle pole.

## Discussion

### Taz1- and microtubule-dependent regulation of centromere detachment from the SPB

**Fig 10:**
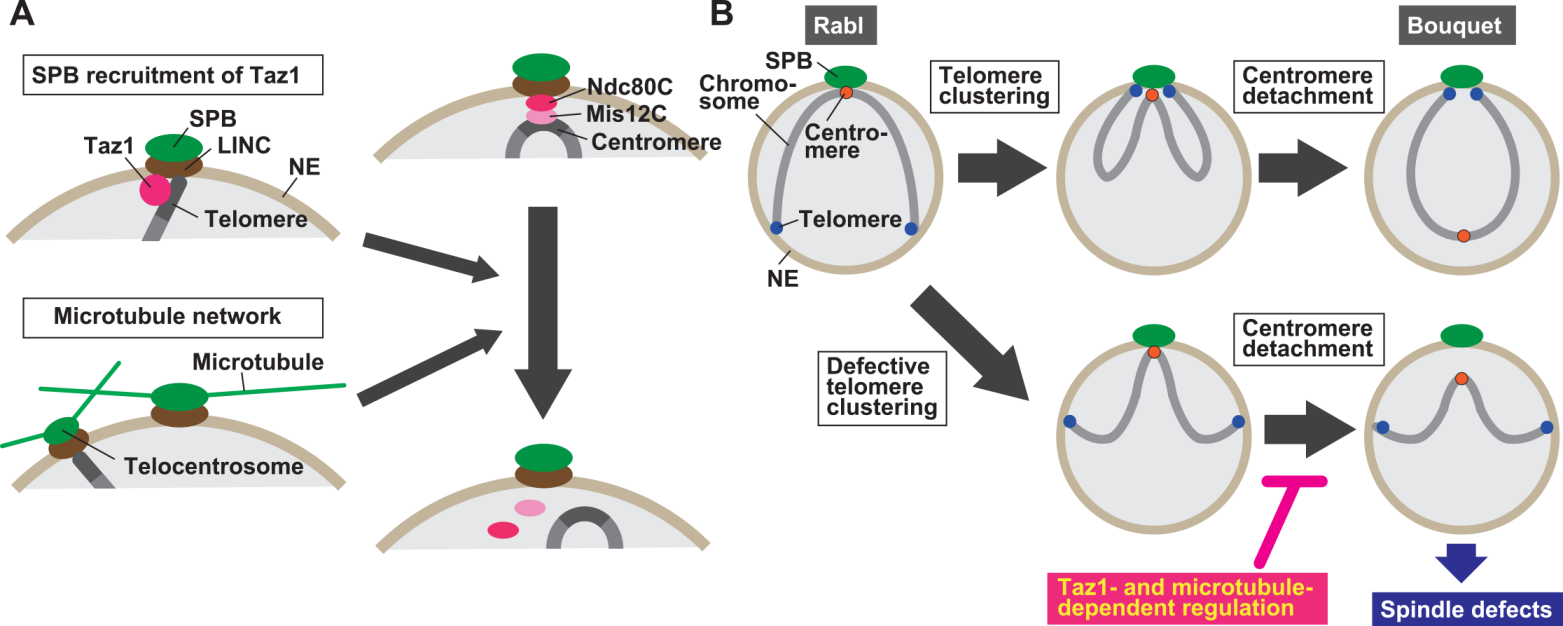
Regulation of centromere detachment from the SPB and its role in meiosis. (A) Regulation of centromere detachment from the SPB. Telomere clustering causes SPB recruitment of Taz1, and SPB-recruited Taz1 promotes centromere detachment from the SPB. SPB- and telocentrosome-nucleated microtubules, which drive telomere clustering, also promote centromere detachment. Ndc80C: Ndc80 complex; Mis12C: Mis12 complex. (B) A role of telomere-dependent regulation of centromere detachment in meiosis. During mitosis centromeres are attached to the SPB, while telomeres are located away from it (Rabl). Centromeres become detached from the SPB after clustering of telomeres at the SPB, forming the bouquet chromosome arrangement (Bouquet). When telomere clustering is impaired, the Taz1- and/or microtubule-dependent regulatory mechanism inhibits centromere detachment, preventing concomitant detachment of centromeres and telomeres from the SPB, which is harmful for spindle formation.

In this study, we showed that kinetochore disassembly-dependent centromere detachment from the SPB is inhibited when telomere clustering is compromised during bouquet formation in *S*. *pombe*. Two lines of evidence show that SPB recruitment of Taz1 promotes centromere detachment (Fig 10A). First, in *taz1Δ* cells, despite occasional SPB association of telomeres, centromere detachment was greatly inhibited. Second, Taz1Δmyb-Sad1 alleviated the inhibition without restoring telomere clustering. We also found that in addition to Taz1, microtubules contribute to centromere detachment (Fig 10A). This conclusion was drawn from the fact that centromere detachment was inhibited by introduction of *mto1Δ* mutation or MBC treatment.

Two facts indicate that the contribution of microtubules is Taz1-independent. First, in cells with a disorganized microtubule network, despite occasional recruitment of Taz1 to the SPB, centromere detachment was inhibited to a level comparable to that in cells in which SPB recruitment of Taz1 was completely defective. Second, Taz1Δmyb-Sad1 failed to restore centromere detachment in *mto1Δ* or MBC-treated cells. Thus, telomere clustering-dependent SPB recruitment of Taz1 and microtubules independently promote centromere detachment in bouquet formation. Taz1- and microtubule-dependent regulatory mechanisms probably inhibit centromere detachment when telomere clustering is impaired.

One likely scenario for Taz1-dependent centromere regulation is that Taz1 recruits a factor(s) to the SPB that promotes centromere detachment by inducing dissociation of outer kinetochore complexes from the centromere and SPB. The promoting factor is perhaps activated or induced by mating pheromone-dependent MAP kinase because, in the absence of mating pheromone signaling, centromeres remain attached to the SPB despite clustering of telomeres [39]. Aurora kinase is a potential candidate for the modifying factor because this kinase induces centromere detachment from the SPB in *S*. *cerevisiae* [60, 61]. Another candidate is Polo-like kinase because it regulates meiotic kinetochore mono-orientation and centromere cohesion [62]. However, it is currently unclear if these kinases contribute to centromere detachment.

Although Taz1 is important, we cannot exclude the possibility that a different telomere-associated factor(s) additionally contributes to centromere detachment. Taz1Δmyb-Sad1 did not appear to completely restore centromere detachment in the telomere clustering-defective mutants (Fig 7C and 7D). Furthermore, the retention level of SPB-centromere association or Nuf2/Mis12 centromere localization in the *taz1Δ* mutant was slightly lower than that in the *bqt1Δ* or *rap1Δ* mutant (Figs 2D and 3C, S3B Fig, Horsetail). These facts may mean the contribution of a different telomere-associated factor to centromere detachment.

How microtubules contribute to centromere detachment remains elusive. Given the requirement of telomere- and/or SPB-nucleated microtubules for telomere clustering, we speculate that efficient centromere detachment requires the telomere/SPB-nucleated microtubules. In *mto1Δ* cells, γ-TuC telomere/SPB localization and telomere/SPB-microtubule interaction are not completely eliminated [45], and the remaining telomere/SPB-nucleated microtubules may account for why inhibition of kinetochore disassembly appeared to be weaker in *mto1Δ* cells than in MBC-treated cells. It is possible that the telomere/SPB-nucleated microtubules recruit a cytoplasmic factor(s) to telomeres and/or the SPB, which activates a centromere detachment-promoting factor in the nucleus, perhaps via the LINC. Alternatively, microtubule-lacking, LINC-accumulated telomeres may generate inhibitory signals for centromere detachment, as spindle-unattached centromeres do for metaphase-anaphase transition in the spindle assembly checkpoint pathway. In either case, the regulatory mechanism is probably different from the Taz1-dependent mechanism because the localization patterns of retained kinetochore components differ between cells lacking telomere-LINC connectors and those defective in microtubule formation (Figs 3D and 4D, S3C Fig).

### Impairment of meiotic centromere formation by telomere-clustering defects

Kinetochore disassembly is thought to be required for establishment of kinetochore mono-orientation and protection of centromere cohesion [39, 40]. Our finding that equational segregation of sister chromatids at meiosis I increased in chiasma-lacking, telomere clustering-defective cells, where kinetochore disassembly is inhibited, supported this idea. In telomere clustering-defective cells, kinetochore mono-orientation is probably compromised because equational segregation is increased in *sgo1Δ rec12Δ* zygotes. It was recently reported that the centromeric histone H3 variant Cnp1 and the heterochromatin protein 1 orthologue Swi6 frequently fail to localize at centromeres in telomere clustering-defective cells [48]. This may mean that kinetochore disassembly also contributes to the proper localization of central centromeric components, and that impaired centromere localization of the central components may cause kinetochore mono-orientation defects. However, if inhibition of kinetochore disassembly induces Cnp1 delocalization, it probably occurs after the horsetail stage because the number of Cnp1 signals was increased during the horsetail stage in the *bqt1Δ* mutant.

### Importance of a regulatory link between centromere detachment and telomere clustering for spindle formation

We showed that Taz1Δmyb-Sad1 induces or increases impairment of spindle formation in telomere clustering-defective *taz1Δ* and *bqt1Δ* cells. Taz1Δmyb-Sad1 induced centromere detachment in these cells; therefore, retention of centromere-SPB association is probably important for spindle formation in the absence of telomere-SPB association. This finding agrees with previous findings that defective telomere clustering causes spindle impairment and that SPB association of centromeres can substitute for SPB association of telomeres and promote spindle formation [34, 35]. Considering these facts together with the strong correlation of the extents of spindle defects with those of defective telomere/centromere-SPB association, we conclude that concurrent detachment of both telomeres and centromeres from the SPB causes spindle impairment, and propose that the Taz1- and microtubule-dependent inhibitory mechanisms of centromere detachment secure proper spindle formation by preventing such harmful detachment (Fig 10B). Microtubule disruption is caused by various environmental factors such as low temperature and osmotic stress; therefore, telomere clustering is probably often inhibited in nature. Despite the inhibition of meiotic centromere formation, cells may have evolved the inhibitory mechanisms of centromere detachment to secure spindle formation.

How telomere/centromere-SPB association contributes to spindle formation is unclear. It was reported that loss of telomere-SPB association leads to decrease in Sad1 at the SPB, and that forced SPB interaction of centromeres restores SPB localization of Sad1 [35]. Given these observations, it was suggested that telomere/centromere-SPB association is required for proper Sad1 localization at the SPB. The decrease in Sad1 at the SPB may account for the dissociation of the SPB from the spindle pole as well as from the nuclear periphery observed in our analyses. Because abolishment of SPB movements restores spindle formation in telomere clustering-defective cells [63], in the absence of telomere/centromere-SPB association, SPB movements may disrupt Sad1 interaction with the nuclear membrane, reducing Sad1 localization at the SPB. Alternatively, defective SPB recruitment of telomere- or centromere-associated factors that regulate Sad1 localization may cause the reduction.

Several facts raise the possibility that similar regulatory mechanisms are present in other organisms. First, meiotic telomere clustering depends on the LINC complexes in other organisms [8, 42, 43]. Second, in mouse oocytes, kinetochore disassembly probably also occurs, because some of the outer kinetochore complexes are not localized at centromeres before meiotic divisions [64]. Third, a telomere-binding protein, Ndj1, which contributes to telomere clustering, regulates meiotic spindle formation by interacting with the SUN-domain protein Mps3 in *S*. *cerevisiae* [18, 19, 65, 66], suggesting the presence of a regulatory relationship between telomeres and spindle formation. Although these facts suggest conservation of the mechanisms, at least in *S*. *cerevisiae*, microtubule-dependent regulation of centromere detachment is apparently missing because microtubule disruption causes centromere detachment from the SPB and induces meiotic centromere formation unlike the situation in *S*. *pombe* [41]. Although conservation of the regulatory mechanisms is currently unclear, there is no doubt that our findings contribute to understanding the mechanisms of meiosis, because telomere and centromere positions play crucial roles in proper meiotic chromosome segregation. Furthermore, chromosome positioning changes dynamically during development and differentiation and contributes to various chromosomal events in many different organisms [1–5], and our findings may also be relevant for understanding the chromosome positioning-dependent mechanisms that regulate development and differentiation of other organisms.

## Materials and Methods

### Yeast strains, media, basic genetic methods, and visualization of intracellular components

The fission yeast strains used in this study are shown in S1 Table. The media and basic genetic manipulation methods used in this study were described by Moreno et al. [67]. The deletion alleles of *bqt1*^*+*^, *rap1*^*+*^, *taz1*^*+*^, and *mto1*^*+*^ were described previously [10, 11, 13, 68]. Visualization of the *sod2*^*+*^ and *cen2* loci, Cnp1, Nuf2, and microtubules was described previously [40, 45, 50, 69, 70]. The fusion gene of *mis12*^*+*^ and GFP was obtained from the Yeast Genetic Resource Center.

Sfi1 was visualized as follows. A DNA fragment encoding mCherry and the P_TET_ terminator was amplified by PCR using the oligonucleotide primers 5´-ACGCGTCGACGAAGATCTTCGGATCCCCGGGTTAATTAAC-3´ and 5´-GGGGTACCATATTACCCTGTTATCCCTAGCG-3´, and an mCherry*-*bearing plasmid as a template, and inserted between the *Sal*I and *Kpn*I sites of an integration vector, pYC36, which transforms *lys1-131* cells into *lys1*^*+*^ cells when it is integrated [71]. The resultant plasmid was digested with *Sma*I and *Sac*II and ligated with a DNA fragment coding the *sfi1*^*+*^ gene and its promoter, which was amplified by PCR using the synthetic oligonucleotide primers, 5´-TCCCCGCGGGGCATTGTATTTGTCAATACCCA-3´ and 5’-AAAGGCCTACGGGTATTAGGAGGTATAGGC-3’, and the fission yeast genomic DNA as a template, and digested with *Sac*II and *Stu*I. The resultant plasmid pJM1 was introduced into *lys1-131* cells, and integrants were selected by the *lys*^*+*^ phenotype. Alternatively, C-terminal mCherry-tagged Sfi1 was generated by the two-step PCR-based method [72]. DNA fragments encoding the Sfi1 C-terminus-coding region or the *sfi1*^*+*^ terminator were amplified by PCR using two sets of synthetic oligonucleotide primers (5´-TTTCAATATTAGTGATTGGAAGCG-3´ and 5´-TTAATTAACCCGGGGATACGGGTATTAGGAGGTATAGGC-3´; 5´-TTTTCGCCTCGACATCATCTCATTTACGATTGACGGAGAGAGT-3´ and 5´-ATACGTATTTCATTTTTGTAAATTTTTC-3´) and genomic DNA as a template. The mCherry module was then amplified by PCR using the two PCR products as primers and pHM22 [45] as a template. The resulting PCR product was introduced into cells, and integrants were selected by resistance to the antibiotic nourseothricin (Werner Bioagents, Jena, Germany) and confirmed by PCR and microscopic observation.

For Mrc1 visualization, a DNA fragment encoding the *mrc1* gene and its promoter was PCR-amplified from fission yeast genomic DNA using oligonucleotide primers, 5´-TCCCCGCGGTCGAAAGGGTACACAAGCGGA-3´ and 5´-AAGGCCTGTCAAAGTCCGAGTAATTATTCAA-3´. The amplified fragment was digested with *Sac*II and *Stu*I and inserted between the *Sac*II and *Sma*I sites of an *mCherry-*coding integration plasmid, pHM4 [45], yielding pAH8, which encodes the Mrc1-mCherry fusion. Mrc1 was visualized by introducing pAH8 into cells.

### Analysis of chromosome positioning and kinetochore complex localization in zygotic meiosis

Cells grown on YES solid medium at 30°C were transferred to ME solid medium and induced to enter meiosis by incubation at 25°C for 14–18 h. Nuclear DNA in meiotic zygotes was stained with DNA-specific Hoechst 33342 dye as described previously [58]. Images of the cells at seven focal planes were taken through a 60×/1.42 NA Plan Apo oil immersion objective lens using an Olympus IX71 inverted microscope (Olympus Corp., Tokyo, Japan) equipped with a cooled charge-coupled device camera (CoolSNAP-HQ2; Nippon Roper Co. Ltd., Tokyo, Japan). Obtained images were processed by deconvolution and analyzed using MetaMorph (version 7) software (Molecular Devices Japan, Inc., Tokyo, Japan).

### Synchronous induction of meiosis in haploid cells and MBC treatment

Haploid cells bearing both mating-type genes were grown in liquid YES medium. They were then induced to enter meiosis by incubation at 30°C in liquid EMM medium lacking a nitrogen source (EMM-N), and treated with MBC, as described previously [45]. For monitoring meiosis progression, 50 μl of the culture was harvested every hour, and nuclear morphology was examined by staining DNA with Hoechst 33342.

### Analysis of sister chromatid segregation at meiosis I

Segregation of sister chromatids in zygotic *rec12* cells was examined as follows. Cells containing the GFP-labeled *cen2* locus and those lacking the GFP-labeled locus of the opposite mating type were grown on YES solid medium at 30°C and mated on ME solid medium at 25°C. For analyzing sister chromatid segregation in haploid meiotic cells, cells containing both mating-type genes were induced to undergo meiosis, as previously described [38, 40]. The meiotic cells were stained with Hoechst 33342, and GFP signals were examined in those containing two chromosomal DNA masses. When analyzing *mto1Δ* zygotes, microtubules were simultaneously visualized by mCherry-tagged *atb2*^*+*^, and only those forming a meiosis-I spindle were examined because two meiosis I-like chromosome masses were frequently formed due to defective karyogamy.

### Expression of an N-terminal portion of Taz1 and its artificial tethering to the SPB

To express an N-terminal portion of Taz1 (Taz1Δmyb), a plasmid bearing a gene encoding Taz1Δmyb was constructed using the integration plasmid pMY23, which encodes an mCherry and *taz1*^*+*^ fusion and the *aur1*^*r*^ gene as a selectable marker [45]. The region that encodes a myb domain of Taz1 in pMY23 was first deleted using the KOD-Plus-Mutagenesis Kit (Toyobo Co., Ltd., Osaka, Japan), generating pJM4. Briefly, a DNA fragment that encodes Taz1Δmyb and mCherry was amplified by PCR using two synthetic oligonucleotide primers, 5’-TTCTCTTCTCAGATTATCACCCTCT-3’ and 5’-AGGATCCCCGGGTTAATTAACAGCA-3’, and pMY23 as a template. After PCR, the template plasmid was removed by *Dpn*I digestion, and the PCR product was circularized by self-ligation, generating pJM4. The sequence of pJM4 was confirmed by DNA sequencing. Then, a DNA fragment encoding the NLS together with a FLAG tag and a Swi6 chromo-domain was amplified by PCR using two synthetic oligonucleotide primers, 5’-GAAGATCTGGATCCTAGTCGCTTTGTTAAAT-3’ and 5’-CCTTAATTAAACCCGGGCCTTTCTTCTTTTTG-3’, and the plasmid Swi6CD-TOPO (a gift from Dr. Jun-ichi Nakayama, Nagoya City University, Nagoya, Aichi, Japan) as a template. The PCR product was digested with *Bgl*II and *Pac*I and placed at the fusion site of Taz1Δmyb and mCherry by inserting it between the *Bam*HI and *Pac*I sites of pJM4. The DNA region encoding a FLAG tag and Swi6 chromo-domain was then removed from the resultant plasmid using the KOD-Plus-Mutagenesis Kit with two synthetic oligonucleotide primers, 5’-CCGCGGGTCGACAGGATCCAAACGGCCT-3’ and 5’-CCTTCTCTTCTCAGATTATCACCCC-3’, as described for pJM4. The resultant plasmid pAW9-1 encodes Taz1Δmyb tagged with NLS and mCherry at its C-terminus. Its sequence was confirmed by DNA sequencing. pAW9-1 was transformed into cells, and integrants were selected by resistance to the antibiotic aureobasidin A (Takara Bio. Inc., Otsu, Japan).

To tether Taz1Δmyb to the SPB, the plasmid pAH16-6, which encodes a fusion of Taz1Δmyb and Sad1, was constructed. pAH16-6 was constructed from a plasmid, pMY35, that encodes Sad1 with the HA epitope tag. pMY35 was constructed as follows. A DNA fragment encoding the HA epitope tag together with the *adh1* terminator Tadh1 was amplified by PCR using synthetic oligonucleotide primers, 5’-ACGCGTCGACGAAGATCTTCGGATCCCCGGGTTAATTAAC-3’ and 5’-GGGGTACCATATTACCCTGTTATCCCTAGCG-3’, and pFA6a-3HA-kanMX6 [73] as a template. The PCR fragment was digested with *Kpn*I and *Sal*I and inserted between the corresponding sites of the integration plasmid pYC36, yielding pTO5. Then, a DNA fragment encoding Sad1 together with its own promoter was amplified by PCR using synthetic oligonucleotide primers, 5’-tccccgcggatgtatccctaacaaacgcaaaaa-3’ and 5’-ccccgctcgagagatgaatcttgacccgtattct-3’, and the fission yeast genomic DNA as a template, and inserted between the *Sac*II and *Sal*I sites of pTO5 after digestion with *Sac*II and *Xho*I. A portion of the resultant plasmid that encodes Sad1 fused with the HA epitope tag was amplified by PCR using synthetic oligonucleotide primers, 5’-CCCAGTCACGACGTTGTAAAAC-3’ and 5’- CCCCGCTCGAGATATTACCCTGTTATCCCTAGCG-3’, digested with *Sac*I and *Xho*I, and inserted between the *Sac*I and *Sal*I sites of the integration plasmid pTO2 bearing the *aur1*^*r*^ gene as a selectable marker [45] to yield pMY35. To construct pAH16-6, a fusion gene of Taz1Δmyb and Sad1 was first constructed by inserting a Taz1Δmyb-encoding DNA fragment into pMY35. A portion of pAW9-1 that encodes Taz1Δmyb and the *taz1*^*+*^ promoter was amplified by PCR using synthetic oligonucleotide primers, 5’-ACGAGCTCATCGACAAGGCATGCGAAGC-3’ and 5’-CCGCTCGAGGATTCTCTTCTCAGATTATCACCC-3’, and inserted between the *Sac*I and *Sal*I sites of pMY35 after digestion with *Sac*I and *Xho*I, yielding the plasmid pAH15-1. Then, pAH16-6 was constructed by replacing a part of pAH15-1 that encodes the HA epitope tag and Tadh1 with an mCherry*-* and T_TET_-coding region of the plasmid pHM4 [45]. The part of pHM4 was amplified by PCR using synthetic oligonucleotide primers, 5’- CCTTAATTAATAGCAAGGGCGAGGAGGATA-3’ and 5’- TCCCCGCGGGGATCTGCCGGTAGAGGT-3’, and inserted between the *Pac*I and *Sac*II sites of pAH15-1 after digestion with the corresponding enzymes, yielding pAH16-6. The sequence of pAH16-6 was confirmed by DNA sequencing. pAH16-6 was transformed into cells, and integrants were selected as described for pAW9-1.

### Live cell analysis of spindle and Nuf2 dynamics

For analysis of spindle dynamics in diploid zygotes, cells were grown on solid YES medium and induced to enter meiosis by incubation for 14–18 h at 25°C on solid ME medium. Then, the cells were suspended in liquid EMM-N medium. For analysis of Nuf2 dynamics, Nuf2-expressing haploid cells bearing both mating-type genes were grown in liquid YES medium and induced to enter meiosis by incubation at 30°C in liquid EMM-N medium, as described previously [45]. For analysis of spindle or Nuf2 dynamics, a drop of the cell suspension was placed on the bottom of 35 mm glass-bottom dishes (Matsunami Glass Ind., Ltd.) coated with 5 mg/ml lectin (Sigma-Aldrich Japan, Inc.). The cells were observed through a 60×/1.42 NA Plan Apo oil immersion objective lens (Olympus Corp.) using a DeltaVision microscope system operated by SoftWoRx software or an IX71 inverted microscope operated by MetaMorph software. Time-lapse images of the cells were collected at eight focal planes spaced at 0.4 μm intervals every 5 or 10 min for spindle dynamics and at nine focal planes spaced at 0.5 μm intervals every 10 min for Nuf2 dynamics using a cooled CCD camera. During collection of time-lapse images, the cells were kept at 25°C. All obtained images were processed by deconvolution, and analyzed using MetaMorph or Priism/IVE software (available at http://www.msg.ucsf.edu/IVE/index.html).

## Supporting Information

S1 Fig Chromosome segregation at meiosis I and a telomere-clustering mechanism. (A) Chromosome segregation and spindle attachment of chromosomes at meiosis I. (B) Contribution of meiosis-specific chromosome properties and the chiasma to sister chromatid co-segregation at meiosis I. Kinetochore mono-orientation and the chiasma facilitate monopolar attachment of sister chromatids, promoting co-segregation of sister chromatids. Persistent centromere cohesion also promotes sister chromatid co-segregation by preventing separation of sister centromeres upon bipolar attachment of sister chromatids. (C) A telomere-clustering mechanism. The telomere-localized LINC complexes form the telocentrosome. Oligomerized, minus end-directed microtubule motors crosslink the telocentrosome- and SPB-nucleated microtubules and gather the telomeres by moving along the microtubules toward the nucleation sites (black arrows). The minus end-directed microtubule motors tethered to the telocentrosome also directly transport the telomeres toward the nucleation sites to aid telomere clustering. Purple arrow: telocentrosome/telomere movement. NE: nuclear envelope.
(EPS)

S2 Fig Centromere localization in *poz1Δ*, *poz1Δ taz1Δ*, and *dhc1Δ* cells during karyogamy. Population of cells containing SPB-associated centromeres. Centromere-SPB association was examined during karyogamy as in Fig 2D. p: p value determined by the Student’s t-test; ns: no significant difference (p>0.05).
(EPS)

S3 Fig Localization of Mis12 in telomere clustering-defective cells. (A) Mis12 localization during mitotic interphase, karyogamy, and the horsetail stage. Arrowheads indicate SPB positions. Karyogamy: karyogamy stage; Horsetail: the horsetail stage. (B) Population of cells containing Mis12 signals. Karyogamy: karyogamy stage; Horsetail: the horsetail stage. Bars show averages of three independent experiments. (C) Population of cells with different Mis12 localization. At SPB: one SPB-associated signal; Off/at SPB: SPB-associated and SPB-dissociated signals; Off SPB: SPB-dissociated signals. Images in the box show the representative localization of the SPB and signals.
(EPS)

S4 Fig Mrc1 localization during meiosis and Nuf2 localization in Mrc1-positive cells. (A) Changes in Mrc1 localization in a zygotic cell. Cells expressing mCherry-tagged Mrc1 (magenta) and GFP-tagged Atb2 (green) were induced to enter meiosis on solid ME medium. They were suspended in EMM-N liquid medium and observed every 15 min under a microscope. Note that the Mrc1 nuclear signal was undetectable shortly after nuclear fusion (45 min), indicating that this signal is an indicator of the early meiotic stage. Numbers indicate time in minutes. Bar: 2 μm. (B) Population of cells with different Nuf2 localization. Horsetail with Mrc1: the horsetail stage with nuclear Mrc1 signals. Numbers in parentheses show the number of examined cells.
(EPS)

S5 Fig Effects of MBC treatment, various mutations, or Taz1Δmyb-Sad1 on meiosis progression of haploid cells. Meiosis progression of haploid cells expressing GFP-tagged Taz1 (A), Cnp1 (B and D), or Nuf2 (C and E). (A, B, and C) Effects of MBC treatment or *bqt1Δ* or *mto1Δ* mutation on haploid meiosis progression. (D and E) Combination effects of MBC treatment, *bqt1Δ* or *mto1Δ* mutation, and Taz1Δmyb-Sad1 on haploid meiosis progression. MBC (+MBC) or DMSO (+DMSO) was added 2 h after nitrogen depletion. More than 100 cells were examined at each time point. 1 nuc: mononuclear cells; 2 nuc: binuclear cells; 2< nuc: cells containing three or four nuclei.
(EPS)

S6 Fig Effects of *moa1Δ* mutation on sister chromatid segregation in chiasma-lacking *sgo1Δ* zygotes. The frequencies of equational segregation of sister chromatids at meiosis I in *rec12Δ sgo1Δ* diploid zygotes. Sister chromatid segregation was analyzed by visualizing the centromere-proximal *lys1* locus of chromosome I [74]. It should be noted that chromosome I undergoes equational segregation more frequently than chromosome II in the *rec12Δ sgo1Δ* background due probably to different centromere structures and/or chromosome lengths [38].
(EPS)

S7 Fig Schematic diagram of the predicted localization of telomere-LINC connectors and Taz1Δmyb fragments and effects of Taz1Δmyb fragments on telomere-SPB association and Nuf2 localization. (A) Predicted localization of telomere-LINC connectors and Taz1Δmyb fragments in various types of cells. In wild-type cells, the telomere-LINC connectors are recruited to the SPB by telomere clustering at the SPB (Wild type). In *taz1Δ* cells, the telomere-LINC connectors other than Taz1 are probably recruited to the SPB by occasional telomere-SPB association. Rap1 interacts with a Sad1 interactor Bqt1 [10]; therefore, telomere-free Rap1 may also interact with the SPB through the Bqt1-Bqt2 complex. Taz1Δmyb likely localizes at the SPB through interaction with Rap1 but not with telomeres in *taz1Δ* cells (*taz1Δ* + Taz1Δmyb). In *bqt1Δ* cells, the telomere-LINC connectors are probably not recruited to the SPB [10], but Taz1Δmyb-Sad1 is probably able to localize at the SPB via Sad1 (*bqt1Δ* + Taz1Δmyb-Sad1). In *mto1Δ* cells, LINC connectors and the LINC complex accumulate at telomeres, but due to defective telomere microtubule nucleation, telomere clustering is defective [45]. Taz1Δmyb-Sad1 is probably localized at telomeres as well as the SPB (*mto1Δ* + Taz1Δmyb-Sad1). Poz1/Tpz1/Pot1: a complex of Poz1, Tpz1, and Pot1; mCh: mCherry molecule. (B) Localization patterns of the telomere-adjacent *sod2* locus during karyogamy in wild-type and telomere clustering-defective cells expressing Taz1Δmyb (+Taz1Δmyb) or Taz1Δmyb-Sad1 (+Taz1Δmyb-Sad1). Numbers in parentheses indicate the number of examined cells. (C) Population of Taz1Δmyb- (+Taz1Δmyb) or Taz1Δmyb-Sad1-expressing (+Taz1Δmyb-Sad1) cells with different Nuf2 localization. At SPB: one SPB-associated signal; Off/at SPB: SPB-associated and SPB-dissociated signals; Off SPB: SPB-dissociated signals. Images in the box show the representative localization of the SPB and signals.
(EPS)

S1 Table Strains used in this study. (DOCX)

S1 Movie Nuf2 localization dynamics during meiosis in a haploid wild-type cell. A haploid wild-type cell bearing both mating-type genes and expressing GFP-tagged Nuf2 was induced to enter meiosis by incubation in EMM-N medium. Images at nine focal planes spaced at 0.5 μm intervals were taken every 10 min, and each set of images was processed by deconvolution and combined to form a maximal projection by Metamorph software. The video plays at a rate of three frames per second.
(MOV)

S2 Movie Nuf2 localization dynamics in relation with the SPB during meiosis in a haploid wild-type cell. This movie is the same as S1 Movie, except that GFP-tagged Nuf2 and mCherry-tagged Sfi1 are simultaneously shown in green and magenta, respectively.
(MOV)

S3 Movie Nuf2 localization dynamics during meiosis in a haploid *bqt1Δ* cell. A haploid *bqt1Δ* cell bearing both mating-type genes and expressing GFP-tagged Nuf2 was induced to enter meiosis by incubation in EMM-N medium. Images at nine focal planes spaced at 0.5 μm intervals were taken every 10 min, and each set of images was processed by deconvolution and combined to form a maximal projection by Metamorph software. The video plays at a rate of three frames per second.
(MOV)

S4 Movie Nuf2 localization dynamics in relation with the SPB during meiosis in a haploid *bqt1Δ* cell. This movie is the same as S3 Movie, except that GFP-tagged Nuf2 and mCherry-tagged Sfi1 are simultaneously shown in green and magenta, respectively.
(MOV)

S5 Movie Dynamics of microtubules, the SPB, and the nuclear membrane during meiosis in a wild-type zygote. Images of a wild-type zygote expressing mCherry-tagged Atb2 and Cut11 (magenta) and GFP-tagged Sid4 (green) were taken at eight focal planes spaced at 0.4 μm intervals every 5 min. Each set of images was processed by deconvolution and combined to form a maximal projection by Priism/IVE software. The video plays at a rate of five frames per second.
(MOV)

S6 Movie Dynamics of microtubules, the SPB, and the nuclear membrane during meiosis in a *bqt1Δ* zygote. Images of a *bqt1Δ* zygote expressing mCherry-tagged Atb2 and Cut11 (magenta) and GFP-tagged Sid4 (green) were taken at eight focal planes spaced at 0.4 μm intervals every 5 min. Each set of images was processed by deconvolution and combined to form a maximal projection by Priism/IVE software. The video plays at a rate of five frames per second.
(MOV)

S7 Movie Dynamics of microtubules, the SPB, and the nuclear membrane during meiosis in a *bqt1Δ* zygote expressing Taz1Δmyb-Sad1. Images of a *bqt1Δ* zygote expressing Taz1Δmyb-Sad1 together with mCherry-tagged Atb2 and Cut11 (magenta) and GFP-tagged Sid4 (green) were taken at eight focal planes spaced at 0.4 μm intervals every 5 min. Each set of images was processed by deconvolution and combined to form a maximal projection by Priism/IVE software. The video plays at a rate of five frames per second.
(MOV)
