# Supplementary material for: A Taz1- and Microtubule-Dependent Regulatory Relationship between Telomere and Centromere Positions in Bouquet Formation Secures Proper Meiotic Divisions
Source: PLoS Genet. 2016 Sep 9;12(9):e1006304. doi: 10.1371/journal.pgen.1006304 (PMC5017736; doi:10.1371/journal.pgen.1006304)
Supplement: S1 Table — (DOCX) [file pgen.1006304.s008.docx]

**S1 Table. Strains used in this study**

| **Figure** | **Strain name** | **Genotype** |
| --- | --- | --- |
| **2A, 2B** | SAH39-13A | *h^–^ leu1-32 ura4-D18 lys1^+^::sfi1^+^-mCherry^(a)^ sod2^+^::kan^r^-ura4^+^-lacO^(b)^ his7^+^::Pdis1-GFP-lacI-NLS^(c)^* |
|  | SAH39-2A | *h^+^ leu1-32 ura4-D18 lys1^+^::sfi1^+^-mCherry sod2^+^::kan^r^-ura4^+^-lacO his7^+^::Pdis1-GFP-lacI-NLS* |
|  | SAW19-2A | *h^–^ leu1-32 ura4-D18 bqt1::LEU2^(d)^ lys1^+^::sfi1^+^-mCherry sod2^+^::kan^r^-ura4^+^-lacO his7^+^::Pdis1-GFP-lacI-NLS* |
|  | SAW14-5C | *h^+^ leu1-32 ura4-D18 bqt1::LEU2 lys1^+^::sfi1^+^-mCherry sod2^+^::kan^r^-ura4^+^-lacO his7^+^::Pdis1-GFP-lacI-NLS* |
|  | SAW29-1B | *h^–^ leu1-32 lys1-131 ura4-D18 taz1::ura4^+(e)^ sfi1^+^::mCherry-nat^r(a)^ sod2^+^::kan^r^-ura4^+^-lacO his7^+^::Pdis1-GFP-lacI-NLS* |
|  | SAW32-4A | *h^+^ leu1-32 lys1-131 ura4-D18 taz1::ura4^+^ sfi1^+^::mCherry-nat^r^ sod2^+^::kan^r^-ura4^+^-lacO his7^+^::Pdis1-GFP-lacI-NLS* |
|  | SAW18-4A | *h^–^ leu1-32 ura4-D18 mto1::kan^r(f)^ lys1^+^::sfi1^+^-mCherry sod2^+^::kan^r^-ura4^+^-lacO his7^+^::Pdis1-GFP-lacI-NLS* |
|  | SAW15-14C | *h^+^ leu1-32 ura4-D18 mto1::kan^r^ lys1^+^::sfi1^+^-mCherry sfi1^+^::mCherry-nat^r^ sod2^+^::kan^r^-ura4^+^-lacO his7^+^::Pdis1-GFP-lacI-NLS* |
| **2B** | SAW41-3B | *h^–^ leu1-32 lys1-131 ura4-D18 rap1::ura4^+(g)^ sfi1^+^::mCherry-nat^r^ sod2^+^::kan^r^-ura4^+^-lacO his7^+^::Pdis1-GFP-lacI-NLS* |
|  | SAW38-22D | *h^+^ leu1-32 lys1-131 ura4-D18 rap1::ura4^+^ sfi1^+^::mCherry-nat^r^ sod2^+^::kan^r^-ura4^+^-lacO his7^+^::Pdis1-GFP-lacI-NLS* |
|  | SKK24-8B | *h^–^ leu1-32 ura4-D18 poz1::ura4^+(h)^ lys1^+^::sfi1^+^-mCherry sod2^+^::kan^r^-ura4^+^-lacO* |
|  | SKK24-14C | *h^+^ leu1-32 ura4-D18 poz1::ura4^+^ lys1^+^::sfi1^+^-mCherry sod2^+^::kan^r^-ura4^+^-lacO his7^+^::Pdis1-GFP-lacI-NLS* |
|  | SKK21-12D | *h^–^ leu1-32 ura4-D18 poz1::ura4^+^ taz1::ura4^+^ lys1^+^::sfi1^+^-mCherry sod2^+^::kan^r^-ura4^+^-lacO* |
|  | SKK21-2C | *h^+^ leu1-32 ura4-D18 poz1::ura4^+^ taz1::ura4^+^ lys1^+^::sfi1^+^-mCherry sod2^+^::kan^r^-ura4^+^-lacO his7^+^::Pdis1-GFP-lacI-NLS* |
| **2C, 2D, 7C** | SJM9-3A | *h^–^ leu1-32 cnp1::nat^r^-Pcnp1-GFP-cnp1^+(i)^ lys1^+^::sfi1^+^-mCherry* |
|  | SJM9-2A | *h^+^ leu1-32 cnp1::nat^r^-Pcnp1-GFP-cnp1^+^ lys1^+^::sfi1^+^-mCherry* |
|  | SJM9-5A | *h^–^ leu1-32 bqt1::LEU2 cnp1::nat^r^-Pcnp1-GFP-cnp1^+^ lys1^+^::sfi1^+^-mCherry* |
|  | SJM9-4A | *h^+^ leu1-32 bqt1::LEU2 cnp1::nat^r^-Pcnp1-GFP-cnp1^+^ lys1^+^::sfi1^+^-mCherry* |
|  | SJM-29-20D | *h^–^ leu1-32 ura4-D18 taz1::ura4^+^ cnp1::nat^r^-Pcnp1-GFP-cnp1^+^ lys1^+^::sfi1^+^-mCherry* |
|  | SEN32-6C | *h^+^ leu1-32 ura4-D18 taz1::ura4^+^ cnp1::nat^r^-Pcnp1-GFP-cnp1^+^ lys1^+^::sfi1^+^-mCherry* |
|  | SJM20-13A | *h^–^ mto1::kan^r^ cnp1::nat^r^-Pcnp1-GFP-cnp1^+^ lys1^+^::sfi1^+^-mCherry* |
|  | SJM20-2A | *h^+^ leu1-32 mto1::kan^r^ cnp1::nat^r^-Pcnp1-GFP-cnp1^+^ lys1^+^::sfi1^+^-mCherry* |
|  | SKTT125-1 | *h^–^ leu1-32 cnp1::nat^r^-Pcnp1-GFP-cnp1^+^ lys1^+^::sfi1^+^-mCherry aur1^+^::aur1^r^-Pnda3-mCherry-atb2^+(j)^* |
|  | SKTT126-1 | *h^+^ leu1-32 cnp1::nat^r^-Pcnp1-GFP-cnp1^+^ lys1^+^::sfi1^+^-mCherry aur1^+^::aur1^r^-Pnda3-mCherry-atb2^+^* |
|  | SKTT143-2 | *h^–^ leu1-32 bqt1::LEU2 cnp1::nat^r^-Pcnp1-GFP-cnp1^+^ lys1^+^::sfi1^+^-mCherry aur1^+^::aur1^r^-Pnda3-mCherry-atb2^+^* |
|  | SKTT144-2 | *h^+^ leu1-32 bqt1::LEU2 cnp1::nat^r^-Pcnp1-GFP-cnp1^+^ lys1^+^::sfi1^+^-mCherry aur1^+^::aur1^r^-Pnda3-mCherry-atb2^+^* |
|  | SKTT128-1 | *h^–^ leu1-32 ura4-D18 taz1::ura4^+^ cnp1::nat^r^-Pcnp1-GFP-cnp1^+^ lys1^+^::sfi1^+^-mCherry aur1^+^::aur1^r^-Pnda3-mCherry-atb2^+^* |
|  | SENT45-1 | *h^+^ leu1-32 ura4-D18 taz1::ura4^+^ cnp1::nat^r^-Pcnp1-GFP-cnp1^+^ lys1^+^::sfi1^+^-mCherry aur1^+^::aur1^r^-Pnda3-mCherry-atb2^+^* |
|  | SKKT21-a | *h^–^ mto1::kan^r^ cnp1::nat^r^-Pcnp1-GFP-cnp1^+^ lys1^+^::sfi1^+^-mCherry aur1^+^::aur1^r^-Pnda3-mCherry-atb2^+^* |
|  | SKKT20-a | *h^+^ leu1-32 mto1::kan^r^ cnp1::nat^r^-Pcnp1-GFP-cnp1^+^ lys1^+^::sfi1^+^-mCherry aur1^+^::aur1^r^-Pnda3-mCherry-atb2^+^* |
| **2D, 7C** | SAH62-2B | *h^–^ leu1-32 ura4-D18 rap1::ura4^+^ cnp1::nat^r^-Pcnp1-GFP-cnp1^+^ lys1^+^::sfi1^+^-mCherry* |
|  | SAH62-3D | *h^+^ leu1-32 ura4-D18 rap1::ura4^+^ cnp1::nat^r^-Pcnp1-GFP-cnp1^+^ lys1^+^::sfi1^+^-mCherry* |
|  | SKKT78-a | *h^–^ leu1-32 ura4-D18 rap1::ura4^+^ cnp1::nat^r^-Pcnp1-GFP-cnp1^+^ lys1^+^::sfi1^+^-mCherry aur1^+^::aur1^r^-Pnda3-mCherry-atb2^+^* |
|  | SKKT77-a | *h^+^ leu1-32 ura4-D18 rap1::ura4^+^ cnp1::nat^r^-Pcnp1-GFP-cnp1^+^ lys1^+^::sfi1^+^-mCherry aur1^+^::aur1^r^-Pnda3-mCherry-atb2^+^* |
| **3B, 3C, 3D, 7D** | SJM24-5D | *h^–^ leu1-32 ura4-D18 nuf2^+^::EGFP-ura4^+(k)^ lys1^+^::sfi1^+^-mCherry* |
|  | SJM18-5C | *h^+^ leu1-32 ura4-D18 nuf2^+^::EGFP-ura4^+^ lys1^+^::sfi1^+^-mCherry* |
|  | SKTT129-1 | *h^–^ leu1-32 ura4-D18 nuf2^+^::EGFP-ura4^+^ lys1^+^::sfi1^+^-mCherry aur1^+^::aur1^r^-Pnda3-mCherry-atb2^+^* |
|  | SKTT130-1 | *h^+^ leu1-32 ura4-D18 nuf2^+^::EGFP-ura4^+^ lys1^+^::sfi1^+^-mCherry aur1^+^::aur1^r^-Pnda3-mCherry-atb2^+^* |
|  | SJM18-7B | *h^–^ leu1-32 ura4-D18 bqt1::LEU2 nuf2^+^::EGFP-ura4^+^ lys1^+^::sfi1^+^-mCherry* |
|  | SJM18-8B | *h^+^ leu1-32 ura4-D18 bqt1::LEU2 nuf2^+^:EGFP-ura4^+^ lys1^+^::sfi1^+^-mCherry* |
|  | SKTT142-1 | *h^–^ leu1-32 ura4-D18 bqt1::LEU2 nuf2^+^:EGFP-ura4^+^ lys1^+^::sfi1^+^-mCherry aur1^+^::aur1^r^-Pnda3-mCherry-atb2^+^* |
|  | SKTT141-1 | *h^+^ leu1-32 ura4-D18 bqt1::LEU2 nuf2^+^:EGFP-ura4^+^ lys1^+^::sfi1^+^-mCherry aur1^+^::aur1^r^-Pnda3-mCherry-atb2^+^* |
|  | SJM29-4C | *h^+^ ade6-M210 leu1-32 ura4-D18 taz1::ura4^+^ nuf2^+^::EGFP-ura4^+^ lys1^+^::sfi1^+^-mCherry* |
|  | SJM29-1D | *h^–^ ade6-M210 leu1-32 ura4-D18 taz1::ura4^+^ nuf2^+^::EGFP-ura4^+^ lys1^+^::sfi1^+^-mCherry* |
|  | SKTT132-1 | *h^–^ ade6-M210 leu1-32 ura4-D18 taz1::ura4^+^ nuf2^+^::EGFP-ura4^+^ lys1^+^::sfi1^+^-mCherry aur1^+^::aur1^r^-Pnda3-mCherry-atb2^+^* |
|  | SKTT131-1 | *h^+^ ade6-M210 leu1-32 ura4-D18 taz1::ura4^+^ nuf2^+^::EGFP-ura4^+^ lys1^+^::sfi1^+^-mCherry aur1^+^::aur1^r^-Pnda3-mCherry-atb2^+^* |
|  | SJM25-9A | *h^–^ leu1-32 ura4-D18 mto1::kan^r^ nuf2^+^::EGFP-ura4^+^ lys1^+^::sfi1^+^-mCherry* |
|  | SJM25-8B | *h^+^ leu1-32 ura4-D18 mto1::kan^r^ nuf2^+^::EGFP-ura4^+^ lys1^+^::sfi1^+^-mCherry* |
|  | SKKT23-a | *h^–^ leu1-32 ura4-D18 mto1::kan^r^ nuf2^+^::EGFP-ura4^+^ lys1^+^::sfi1^+^-mCherry aur1^+^::aur1^r^-Pnda3-mCherry-atb2^+^* |
|  | SKKT22-a | *h^+^ leu1-32 ura4-D18 mto1::kan^r^ nuf2^+^::EGFP-ura4^+^ lys1^+^::sfi1^+^-mCherry aur1^+^::aur1^r^-Pnda3-mCherry-atb2^+^* |
| **3C, 3D, 7D** | SAWT50-1 | *h^–^ leu1-32 ura4-D18 lys1^+^::sfi1^+^-mCherry rap1::ura4^+^ nuf2^+^::EGFP-ura4^+^* |
|  | SAWT49-1 | *h^+^ leu1-32 ura4-D18 lys1^+^::sfi1^+^-mCherry rap1::ura4^+^ nuf2^+^::EGFP-ura4^+^* |
|  | SKKT80-a | *h^–^ leu1-32 ura4-D18 rap1::ura4^+^ nuf2^+^::EGFP-ura4^+^ lys1^+^::sfi1^+^-mCherry aur1^+^::aur1^r^-Pnda3-mCherry-atb2^+^* |
|  | SKKT79-a | *h^+^ leu1-32 ura4-D18 rap1::ura4^+^ nuf2^+^::EGFP-ura4^+^ lys1^+^::sfi1^+^-mCherry aur1^+^::aur1^r^-Pnda3-mCherry-atb2^+^* |
| **3C, S4B** | SAHT51-13 | *h^—^ leu1-32 ura4-D18 nuf2^+^::EGFP-ura4^+^ lys1^+^::sfi1^+^-mCherry aur1^+^::aur1^r^-mrc1-mCherry^(a)^* |
|  | SAHT48-2 | *h^+^ leu1-32 ura4-D18 nuf2^+^::EGFP-ura4^+^ lys1^+^::sfi1^+^-mCherry aur1^+^::aur1^r^-mrc1-mCherry* |
|  | SAHT84-1 | *h^—^ leu1-32 ura4-D18 bqt1::LEU2 nuf2^+^::EGFP-ura4^+^ lys1^+^::sfi1^+^-mCherry aur1^+^::aur1^r^-mrc1-mCherry* |
|  | SAHT85-8 | *h^+^ leu1-32 ura4-D18 bqt1::LEU2 nuf2^+^::EGFP-ura4^+^ lys1^+^::sfi1^+^-mCherry aur1^+^::aur1^r^-mrc1-mCherry* |
|  | SAHT54-1 | *h^—^ ade6-M210 leu1-32 ura4-D18 taz1::ura4^+^ nuf2^+^::EGFP-ura4^+^ lys1^+^::sfi1^+^-mCherry aur1^+^::aur1^r^-mrc1-mCherry* |
|  | SAHT55-3 | *h^+^ ade6-M210 leu1-32 ura4-D18 taz1::ura4^+^ nuf2^+^::EGFP-ura4^+^ lys1^+^::sfi1^+^-mCherry aur1^+^::aur1^r^-mrc1-mCherry* |
|  | SAHT53-2 | *h^—^ leu1-32 ura4-D18 mto1::kan^r^ nuf2^+^::EGFP-ura4^+^ lys1^+^::sfi1^+^-mCherry aur1^+^::aur1^r^-mrc1-mCherry* |
|  | SAHT52-3 | *h^+^ leu1-32 ura4-D18 mto1::kan^r^ nuf2^+^::EGFP-ura4^+^ lys1^+^::sfi1^+^-mCherry aur1^+^::aur1^r^-mrc1-mCherry* |
| **S3** | SKKT2-2A | *h^–^ leu1-32 ura4-D18 mis12^+^::GFP-LEU2^(g)^ lys1^+^::sfi1^+^-mCherry* |
|  | SKKT1-2C | *h^+^ leu1-32 ura4-D18 mis12^+^::GFP-LEU2 lys1^+^::sfi1^+^-mCherry* |
|  | SKTT137-1 | *h^–^ leu1-32 ura4-D18 mis12^+^::GFP-LEU2 lys1^+^::sfi1^+^-mCherry aur1^+^::aur1^r^-Pnda3-mCherry-atb2^+^* |
|  | SKTT138-1 | *h^+^ leu1-32 ura4-D18 mis12^+^::GFP-LEU2 lys1^+^::sfi1^+^-mCherry aur1^+^::aur1^r^-Pnda3-mCherry-atb2^+^* |
|  | SKT68-4D | *h^–^ leu1-32 bqt1::LEU2 mis12^+^::GFP-LEU2 lys1^+^::sfi1^+^-mCherry* |
|  | SKT68-1C | *h^+^ leu1-32 bqt1::LEU2 mis12^+^::GFP-LEU2 lys1^+^::sfi1^+^-mCherry* |
|  | SKTT150-1 | *h^–^ leu1-32 bqt1::LEU2 mis12^+^::GFP-LEU2 lys1^+^::sfi1^+^-mCherry aur1^+^::aur1^r^-Pnda3-mCherry-atb2^+^* |
|  | SKTT149-1 | *h^+^ leu1-32 bqt1::LEU2 mis12^+^::GFP-LEU2 lys1^+^::sfi1^+^-mCherry aur1^+^::aur1^r^-Pnda3-mCherry-atb2^+^* |
|  | SKK5-3A | *h^–^ leu1-32 ura4-D18 taz1::ura4^+^ mis12^+^::GFP-LEU2 lys1^+^::sfi1^+^-mCherry* |
|  | SKK5-5C | *h^+^ leu1-32 ura4-D18 taz1::ura4^+^ mis12^+^::GFP-LEU2 lys1^+^::sfi1^+^-mCherry* |
|  | SKTT140-1 | *h^–^ leu1-32 ura4-D18 taz1::ura4^+^ mis12^+^::GFP-LEU2 lys1^+^::sfi1^+^-mCherry aur1^+^::aur1^r^-Pnda3-mCherry-atb2^+^* |
|  | SKTT139-1 | *h^+^ leu1-32 ura4-D18 taz1::ura4^+^ mis12^+^::GFP-LEU2 lys1^+^::sfi1^+^-mCherry aur1^+^::aur1^r^-Pnda3-mCherry-atb2^+^* |
|  | SKKT3-3B | *h^–^ leu1-32 ura4-D18 mto1::kan^r^ mis12^+^::GFP-LEU2 lys1^+^::sfi1^+^-mCherry* |
|  | SKKT4-2A | *h^+^ leu1-32 ura4-D18 mto1::kan^r^ mis12^+^::GFP-LEU2 lys1^+^::sfi1^+^-mCherry* |
|  | SKKT25-a | *h^–^ leu1-32 ura4-D18 mto1::kan^r^ mis12^+^::GFP-LEU2 lys1^+^::sfi1^+^-mCherry aur1^+^::aur1^r^-Pnda3-mCherry-atb2^+^* |
|  | SKKT24-a | *h^+^ leu1-32 ura4-D18 mto1::kan^r^ mis12^+^::GFP-LEU2 lys1^+^::sfi1^+^-mCherry aur1^+^::aur1^r^-Pnda3-mCherry-atb2^+^* |
| **3E** | SAH40-1A | *h^–^ leu1-32 lys1-131 ura4-D18 taz1::ura4^+^ nuf2^+^::mRFP-ura4^+(k)^ cnp1::nat^r^-Pcnp1-GFP-cnp1^+^* |
|  | SAH40-6C | *h^+^ leu1-32 lys1-131 ura4-D18 taz1::ura4^+^ nuf2^+^::mRFP-ura4^+^ cnp1::nat^r^-Pcnp1-GFP-cnp1^+^* |
| **S4A** | SAHT45-1 | *h^90^ ade6-M210 leu1-32 ura4-D18 lys1^+^::Pnda3-GFP-atb2^+^ aur1^+^::aur1^r^-mrc1-mCherry* |
| **4A, S5A** | SMYT144-2 | *h^–^ ura4-D18 taz1::ura4^+^ lys1^+^::taz1^+^-GFP^(l)^ sid4^+^::mRFP-kan^r(j)^ aur1^+^::aur1^r^-mat-P^(j)^* |
|  | SKK58-5A | *h^–^ leu1-32 taz1^+^::GFP-kan^r^ sfi1^+^::mCherry-nat^r^ bqt1::LEU2 lys1^+^::mat-P^(m)^* |
|  | SEN13-5B | *h^–^ taz1^+^::GFP-kan^r^ sfi1^+^::mCherry-nat^r^ mto1::kan^r^ lys1^+^::mat-P* |
| **4B, 4C, S5B** | SKTT95-1 | *h^–^ cnp1::nat^r^-Pcnp1-GFP-cnp1^+^ lys1^+^::sfi1^+^-mCherry aur1^+^::aur1^r^-mat-P* |
| **4B, 4C, 8A, S5B,** | SKK55-12B | *h^–^ cnp1::nat^r^-Pcnp1-GFP-cnp1^+^ sfi1^+^::mCherry-nat^r^ mto1::kan^r^ lys1^+^::mat-P* |
| **4B, 4C, 8B, S5B, S5D** | SKKT184-1 | *h^–^ leu1-32 cnp1::nat^r^-Pcnp1-GFP-cnp1^+^ sfi1^+^::mCherry-nat^r^ bqt1::LEU2 lys1^+^::mat-P* |
| **4D, 4E, 5, 8A, S5C** | SKK53-3A | *h^–^ ura4-D18 nuf2^+^::EGFP-ura4^+^ sfi1^+^::mCherry-nat^r^ lys1^+^::mat-P* |
| **4D, 4E, 8A, S5C** | SKK56-8B | *h^–^ ura4-D18 nuf2^+^::EGFP-ura4^+^ sfi1^+^::mCherry-nat^r^ mto1::kan^r^ lys1^+^::mat-P* |
| **4D, 4E, 5, 8B, S5C, S5E** | SKK48-17B | *h^–^ leu1-32? ura4-D18 nuf2^+^::EGFP-ura4^+^ sfi1^+^::mCherry-nat^r^ bqt1::LEU2 lys1^+^::mat-P* |
| **6B** | SY15-26 | *h^+^ leu1-32 lys1-131 rec12-152::LEU2^(n)^ his7^+^::Pdis1-GFP-lacI-NLS* |
|  | SY16-43 | *h^–^ leu1-32 lys1-131 ura4-D18 rec12-152::LEU2 cen2(D107)::kan^r^-ura4^+^-lacO^(m)^ his7^+^::Pdis1-GFP-lacI-NLS* |
|  | SAH31-11C | *h^—^ leu1-32 lys1-131 ura4-D18 bqt1::LEU2 rec12-152::LEU2 cen2(D107)::kan^r^-ura4^+^-lacO his7^+^::Pdis1-GFP-lacI-NLS* |
|  | SAH31-11A | *h^+^ leu1-32 lys1-131 ura4-D18 bqt1::LEU2 rec12-152::LEU2 his7^+^::Pdis1-GFP-lacI-NLS* |
|  | SAH43-17A | *h^—^ leu1-32 lys1-131 ura4-D18 mto1::kan^r^ rec12-152::LEU2 his7^+^::Pdis1-GFP-lacI-NLS* |
|  | SAH43-2A | *h^+^ leu1-32 ura4-D18 mto1::kan^r^ rec12-152::LEU2 lys1^+^::Pnda3-GFP-atb2^+^ cen2(D107)::kan^r^-ura4^+^-lacO his7^+^::Pdis1-GFP-lacI-NLS* |
| **6C** | SYH18-2-6D | *h^—^ leu1-32 ura4-D18 sgo1::kan^r(o)^ rec12-152::LEU2 his7^+^::Pdis1-GFP-lacI-NLS* |
|  | SYH18-2-1C | *h^+^ leu1-32 lys1-131 ura4-D18 sgo1::kan^r^ rec12-152::LEU2 cen2(D107)::kan^r^-ura4^+^-lacO his7^+^::Pdis1-GFP-lacI-NLS* |
|  | SAH31-10A | *h^—^ leu1-32 lys1-131 ura4-D18 bqt1::LEU2 sgo1::kan^r^ rec12-152::LEU2 cen2(D107)::kan^r^-ura4-lacO his7^+^::Pdis1-GFP-lacI-NLS* |
|  | SAH12-4B | *h^+^ leu1-32 lys1-131 ura4-D18 bqt1::LEU2 sgo1::kan^r^ rec12-152::LEU2 his7^+^::Pdis1-GFP-lacI-NLS* |
| **6E, 6F** | SAHT115-1 | *h^—^ ura4-D18 lys1^+^::mat-P cen2(D107)::kan^r^-ura4^+^-lacO his7^+^::Pdis1-GFP-lacI-NLS* |
| **6E** | SAHT109-2 | *h^—^ leu1-32 ura4-D18 bqt1::LEU2 lys1^+^::mat-P cen2(D107)::kan^r^-ura4-lacO his7^+^::Pdis1-GFP-lacI-NLS* |
| **S6** | SYH91-15B | *h^+^ leu1-32 ura4-D18 lys1^+^::lacO^(c)^ sgo1::kan^r^ rec12-152::LEU2 his7^+^::Pdis1-GFP-lacI-NLS* |
|  | SYH91-1C | *h^—^ leu1-32 ura4-D18 sgo1::kan^r^ rec12-152::LEU2 his7^+^::Pdis1-GFP-lacI-NLS* |
|  | SYH86-6D | *h^+^ leu1-32 ura4-D18 moa1::kan^r(p)^ lys1^+^::lacO sgo1::kan^r^ rec12-152::LEU2 his7^+^::Pdis1-GFP-lacI-NLS* |
|  | SYH145-16B | *h^—^ leu1-32 ura4-D18 moa1::kan^r^ sgo1::kan^r^ rec12-152::LEU2 his7^+^::Pdis1-GFP-lacI-NLS* |
| **7B** | SAWT10-2 | *h^90^ leu1-32 sid4^+^::GFP-kan^r(q)^ aur1^+^::aur1^r^-Ptaz1-taz1∆myb-NLS-mCherry^(a)^* |
|  | SKK15-8D | *h^—^ leu1-32 lys1-131 bqt1::LEU2 sid4^+^::GFP-kan^r^ aur1^+^::aur1^r^-Ptaz1-taz1∆myb-NLS-mCherry* |
|  | SKK15-1B | *h^+^ leu1-32 lys1-131 bqt1::LEU2 sid4^+^::GFP-kan^r^ aur1^+^::aur1^r^-Ptaz1-taz1∆myb-NLS-mCherry* |
|  | SKKT28-a | *h^—^ leu1-32 lys1-131 taz1::ura4^+^ sid4^+^::GFP-kan^r^ aur1^+^::aur1^r^-Ptaz1-taz1∆myb-NLS-mCherry* |
|  | SKKT27-a | *h^+^ leu1-32 lys1-131 ura4? taz1::ura4^+^ sid4^+^::GFP-kan^r^ aur1^+^::aur1^r^-Ptaz1-taz1∆myb-NLS-mCherry* |
|  | SAHT90-5 | *h^—^ leu1-32 lys1-131 ura4-D18 mto1::kan^r^ sid4^+^::GFP-kan^r^ aur1^+^::aur1^r^-Ptaz1-taz1∆myb-NLS-mCherry* |
|  | SKKT92-a | *h^+^ his2-245 leu1-32 lys1-131 ura4-D18 mto1::kan^r^ sid4^+^::GFP-kan^r^ aur1^+^::aur1^r^-Ptaz1-taz1∆myb-NLS-mCherry* |
|  | SAHT143-3 | *h^90^ leu1-32 sid4^+^::GFP-kan^r^ aur1^+^::aur1^r^-Ptaz1-taz1∆myb-sad1-mCherry^(a)^* |
|  | SAHT147-1 | *h^—^ ade6-M210 leu1-32 lys1-131 bqt1::LEU2 sid4^+^::GFP-kan^r^ aur1^+^::aur1^r^-Ptaz1-taz1∆myb-sad1-mCherry* |
|  | SAHT146-1 | *h^+^ ade6-M210 leu1-32 lys1-131 bqt1::LEU2 sid4^+^::GFP-kan^r^ aur1^+^::aur1^r^-Ptaz1-taz1∆myb-sad1-mCherry* |
|  | SAHT203-2 | *h^—^ leu1-32 lys1-131 ura4-D18 taz1::ura4^+^ sid4^+^::GFP-kan^r^ aur1^+^::aur1^r^-Ptaz1-taz1∆myb-sad1-mCherry* |
|  | SKKT76-a | *h^+^ leu1-32 lys1-131 ura4-D18 taz1::ura4^+^ sid4^+^::GFP-kan^r^ aur1^+^::aur1^r^-Ptaz1-taz1∆myb-sad1-mCherry* |
|  | SKKT90-a | *h^—^ leu1-32 lys1-131 ura4-D18 mto1::kan^r^ sid4^+^::GFP-kan^r^ aur1^+^::aur1^r^-Ptaz1-taz1∆myb-sad1-mCherry* |
|  | SKKT89-a | *h^+^ his2-245 leu1-32 lys1-131 ura4-D18 mto1::kan^r^ sid4^+^::GFP-kan^r^ aur1^+^::aur1^r^-Ptaz1-taz1∆myb-sad1-mCherry* |
| **S7B** | SAHT102-1 | *h^–^ leu1-32 ura4-D18 lys1^+^::sfi1^+^-mCherry sod2^+^::kan^r^-ura4^+^-lacO his7^+^::Pdis1-GFP-lacI-NLS aur1^+^::aur1^r^-Ptaz1-taz1∆myb-NLS-mCherry* |
|  | SAHT101-7 | *h^+^ leu1-32 ura4-D18 lys1^+^::sfi1^+^-mCherry sod2^+^::kan^r^-ura4^+^-lacO his7^+^::Pdis1-GFP-lacI-NLS aur1^+^::aur1^r^-Ptaz1-taz1∆myb-NLS-mCherry* |
|  | SAW16-7B | *h^–^ leu1-32 ura4-D18 bqt1::LEU2 lys1^+^::sfi1^+^-mCherry sod2^+^::kan^r^-ura4^+^-lacO his7^+^::Pdis1-GFP-lacI-NLS aur1^+^::aur1^r^-Ptaz1-taz1∆myb-NLS-mCherry* |
|  | SAW14-7A | *h^+^ leu1-32 ura4-D18 bqt1::LEU2 lys1^+^::sfi1^+^-mCherry sod2^+^::kan^r^-ura4^+^-lacO his7^+^::Pdis1-GFP-lacI-NLS aur1^+^::aur1^r^-Ptaz1-taz1∆myb-NLS-mCherry* |
|  | SAHT200-7 | *h^–^ leu1-32 lys1-131 ura4-D18 rap1::ura4^+^ sfi1^+^::mCherry-nat^r^ sod2^+^::kan^r^-ura4^+^-lacO his7^+^::Pdis1-GFP-lacI-NLS aur1^+^::aur1^r^-Ptaz1-taz1∆myb-NLS-mCherry* |
|  | SAHT199-8 | *h^+^ leu1-32 lys1-131 ura4-D18 rap1::ura4^+^ sfi1^+^::mCherry-nat^r^ sod2^+^::kan^r^-ura4^+^-lacO his7^+^::Pdis1-GFP-lacI-NLS aur1^+^::aur1^r^-Ptaz1-taz1∆myb-NLS-mCherry* |
|  | SAHT99-2 | *h^–^ leu1-32 ura4-D18 taz1::ura4^+^ lys1^+^::sfi1^+^-mCherry sod2^+^::kan^r^-ura4^+^-lacO his7^+^::Pdis1-GFP-lacI-NLS aur1^+^::aur1^r^-Ptaz1-taz1∆myb-NLS-mCherry* |
|  | SAHT100-3 | *h^+^ leu1-32 ura4-D18 taz1::ura4^+^ lys1^+^::sfi1^+^-mCherry sod2^+^::kan^r^-ura4^+^-lacO his7^+^::Pdis1-GFP-lacI-NLS aur1^+^::aur1^r^-Ptaz1-taz1∆myb-NLS-mCherry* |
|  | SAW17-13B | *h^–^ leu1-32 ura4-D18 mto1::kan^r^ lys1^+^::sfi1^+^-mCherry sod2^+^::kan^r^-ura4^+^-lacO his7^+^::Pdis1-GFP-lacI-NLS aur1^+^::aur1^r^-Ptaz1-taz1∆myb-NLS-mCherry* |
|  | SAW15-8B | *h^+^ leu1-32 ura4-D18 mto1::kan^r^ lys1^+^::sfi1^+^-mCherry sod2^+^::kan^r^-ura4^+^-lacO his7^+^::Pdis1-GFP-lacI-NLS aur1^+^::aur1^r^-Ptaz1-taz1∆myb-NLS-mCherry* |
|  | SAHT159-1 | *h^–^ leu1-32 ura4-D18 lys1^+^::sfi1^+^-mCherry sod2^+^::kan^r^-ura4^+^-lacO his7^+^::Pdis1-GFP-lacI-NLS aur1^+^::aur1^r^-Ptaz1-taz1∆myb-sad1-mCherry* |
|  | SAHT158-1 | *h^+^ leu1-32 ura4-D18 lys1^+^::sfi1^+^-mCherry sod2^+^::kan^r^-ura4^+^-lacO his7^+^::Pdis1-GFP-lacI-NLS aur1^+^::aur1^r^-Ptaz1-taz1∆myb-sad1-mCherry* |
|  | SAHT161-1 | *h^–^ leu1-32 ura4-D18 bqt1::LEU2 lys1^+^::sfi1^+^-mCherry sod2^+^::kan^r^-ura4^+^-lacO his7^+^::Pdis1-GFP-lacI-NLS aur1^+^::aur1^r^-Ptaz1-taz1∆myb-sad1-mCherry* |
|  | SAHT160-1 | *h^+^ leu1-32 ura4-D18 bqt1::LEU2 lys1^+^::sfi1^+^-mCherry sod2^+^::kan^r^-ura4^+^-lacO his7^+^::Pdis1-GFP-lacI-NLS aur1^+^::aur1^r^-Ptaz1-taz1∆myb-sad1-mCherry* |
|  | SAHT202-1 | *h^–^ leu1-32 lys1-131 ura4-D18 rap1::ura4^+^ sfi1^+^::mCherry-nat^r^ sod2^+^::kan^r^-ura4^+^-lacO his7^+^::Pdis1-GFP-lacI-NLS aur1^+^::aur1^r^-Ptaz1-taz1∆myb-sad1-mCherry* |
|  | SAHT201-1 | *h^+^ leu1-32 lys1-131 ura4-D18 rap1::ura4^+^ sfi1^+^::mCherry-nat^r^ sod2^+^::kan^r^-ura4^+^-lacO his7^+^::Pdis1-GFP-lacI-NLS aur1^+^::aur1^r^-Ptaz1-taz1∆myb-sad1-mCherry* |
|  | SAHT178-1 | *h^–^ leu1-32 lys1-131 ura4-D18 taz1::ura4^+^ sfi1^+^::mCherry-nat^r^ sod2^+^::kan^r^-ura4^+^-lacO his7^+^::Pdis1-GFP-lacI-NLS aur1^+^::aur1^r^-Ptaz1-taz1∆myb-sad1-mCherry* |
|  | SAHT179-2 | *h^+^ leu1-32 lys1-131 ura4-D18 taz1::ura4^+^ sfi1^+^::mCherry-nat^r^ sod2^+^::kan^r^-ura4^+^-lacO his7^+^::Pdis1-GFP-lacI-NLS aur1^+^::aur1^r^-Ptaz1-taz1∆myb-sad1-mCherry* |
|  | SAHT163-1 | *h^–^ leu1-32 ura4-D18 mto1::kan^r^ lys1^+^::sfi1^+^-mCherry sod2^+^::kan^r^-ura4^+^-lacO his7^+^::Pdis1-GFP-lacI-NLS aur1^+^::aur1^r^-Ptaz1-taz1∆myb-sad1-mCherry* |
|  | SAHT162-1 | *h^+^ leu1-32 ura4-D18 mto1::kan^r^ lys1^+^::sfi1^+^-mCherry sod2^+^::kan^r^-ura4^+^-lacO his7^+^::Pdis1-GFP-lacI-NLS aur1^+^::aur1^r^-Ptaz1-taz1∆myb-sad1-mCherry* |
| **7C** | SKTT134-1 | *h^—^ leu1-32 cnp1::nat^r^-Pcnp1-GFP-cnp1^+^ lys1^+^::sfi1^+^-mCherry aur1^+^::aur1^r^-Ptaz1-taz1∆myb-NLS-mCherry* |
|  | SKTT133-1 | *h^+^ leu1-32 cnp1::nat^r^-Pcnp1-GFP-cnp1^+^ lys1^+^::sfi1^+^-mCherry aur1^+^::aur1^r^-Ptaz1-taz1∆myb-NLS-mCherry* |
|  | SKTT145-1 | *h^—^ leu1-32 bqt1::LEU2 cnp1::nat^r^-Pcnp1-GFP-cnp1^+^ lys1^+^::sfi1^+^-mCherry aur1^+^::aur1^r^-Ptaz1-taz1∆myb-NLS-mCherry* |
|  | SKTT146-1 | *h^+^ leu1-32 bqt1::LEU2 cnp1::nat^r^-Pcnp1-GFP-cnp1^+^ lys1^+^::sfi1^+^-mCherry aur1^+^::aur1^r^-Ptaz1-taz1∆myb-NLS-mCherry* |
|  | SKKT86-a | *h^—^ leu1-32 ura4-D18 rap1::ura4^+^ cnp1::nat^r^-Pcnp1-GFP-cnp1^+^ lys1^+^::sfi1^+^-mCherry aur1^+^::aur1^r^-Ptaz1-taz1∆myb-NLS-mCherry* |
|  | SKKT85-a | *h^+^ leu1-32 ura4-D18 rap1::ura4^+^ cnp1::nat^r^-Pcnp1-GFP-cnp1^+^ lys1^+^::sfi1^+^-mCherry aur1^+^::aur1^r^-Ptaz1-taz1∆myb-NLS-mCherry* |
|  | SKTT135-1 | *h^—^ leu1-32 ura4-D18 taz1::ura4^+^ cnp1::nat^r^-Pcnp1-GFP-cnp1^+^ lys1^+^::sfi1^+^-mCherry aur1^+^::aur1^r^-Ptaz1-taz1∆myb-NLS-mCherry* |
|  | SENT43-1 | *h^+^ leu1-32 ura4-D18 taz1::ura4^+^ cnp1::nat^r^-Pcnp1-GFP-cnp1^+^ lys1^+^::sfi1^+^-mCherry aur1^+^::aur1^r^-Ptaz1-taz1∆myb-NLS-mCherry* |
|  | SKKT30-a | *h^—^ mto1::kan^r^ cnp1::nat^r^-Pcnp1-GFP-cnp1^+^ lys1^+^::sfi1^+^-mCherry aur1^+^::aur1^r^-Ptaz1-taz1∆myb-NLS-mCherry* |
|  | SKKT29-a | *h^+^ leu1-32 mto1::kan^r^ cnp1::nat^r^-Pcnp1-GFP-cnp1^+^ lys1^+^::sfi1^+^-mCherry aur1^+^::aur1^r^-Ptaz1-taz1∆myb-NLS-mCherry* |
|  | SAHT151-1 | *h^—^ leu1-32 cnp1::nat^r^-Pcnp1-GFP-cnp1^+^ lys1^+^::sfi1^+^-mCherry aur1^+^::aur1^r^-Ptaz1-taz1∆myb-sad1-mCherry* |
|  | SAHT150-2 | *h^+^ leu1-32 cnp1::nat^r^-Pcnp1-GFP-cnp1^+^ lys1^+^::sfi1^+^-mCherry aur1^+^::aur1^r^-Ptaz1-taz1∆myb-sad1-mCherry* |
|  | SAHT153-1 | *h^—^ leu1-32 bqt1::LEU2 cnp1::nat^r^-Pcnp1-GFP-cnp1^+^ lys1^+^::sfi1^+^-mCherry aur1^+^::aur1^r^-Ptaz1-taz1∆myb-sad1-mCherry* |
|  | SAHT152-1 | *h^+^ leu1-32 bqt1::LEU2 cnp1::nat^r^-Pcnp1-GFP-cnp1^+^ lys1^+^::sfi1^+^-mCherry aur1^+^::aur1^r^-Ptaz1-taz1∆myb-sad1-mCherry* |
|  | SKKT82-a | *h^—^ leu1-32 ura4-D18 rap1::ura4^+^ cnp1::nat^r^-Pcnp1-GFP-cnp1^+^ lys1^+^::sfi1^+^-mCherry aur1^+^::aur1^r^-Ptaz1-taz1∆myb-sad1-mCherry* |
|  | SKKT81-a | *h^+^ leu1-32 ura4-D18 rap1::ura4^+^ cnp1::nat^r^-Pcnp1-GFP-cnp1^+^ lys1^+^::sfi1^+^-mCherry aur1^+^::aur1^r^-Ptaz1-taz1∆myb-sad1-mCherry* |
|  | SAHT175-1 | *h^—^ leu1-32 ura4-D18 taz1::ura4^+^ cnp1::nat^r^-Pcnp1-GFP-cnp1^+^ lys1^+^::sfi1^+^-mCherry aur1^+^::aur1^r^-Ptaz1-taz1∆myb-sad1-mCherry* |
|  | SENT44-1 | *h^+^ leu1-32 ura4-D18 taz1::ura4^+^ cnp1::nat^r^-Pcnp1-GFP-cnp1^+^ lys1^+^::sfi1^+^-mCherry aur1^+^::aur1^r^-Ptaz1-taz1∆myb-sad1-mCherry* |
|  | SAHT155-1 | *h^—^ mto1::kan^r^ cnp1::nat^r^-Pcnp1-GFP-cnp1^+^ lys1^+^::sfi1^+^-mCherry aur1^+^::aur1^r^-Ptaz1-taz1∆myb-sad1-mCherry* |
|  | SAHT154-1 | *h^+^ leu1-32 mto1::kan^r^ cnp1::nat^r^-Pcnp1-GFP-cnp1^+^ lys1^+^::sfi1^+^-mCherry aur1^+^::aur1^r^-Ptaz1-taz1∆myb-sad1-mCherry* |
| **7D, S7C** | SAHT58-2 | *h^—^ leu1-32 ura4-D18 nuf2^+^::EGFP-ura4^+^ lys1^+^::sfi1^+^-mCherry aur1^+^::aur1^r^-Ptaz1-taz1∆myb-NLS-mCherry* |
|  | SAHT59-11 | *h^+^ leu1-32 ura4-D18 nuf2^+^::EGFP-ura4^+^ lys1^+^::sfi1^+^-mCherry aur1^+^::aur1^r^-Ptaz1-taz1∆myb-NLS-mCherry* |
|  | SAHT56-1 | *h^—^ leu1-32 ura4-D18 bqt1::LEU2 nuf2^+^::EGFP-ura4^+^ lys1^+^::sfi1^+^-mCherry aur1^+^::aur1^r^-Ptaz1-taz1∆myb-NLS-mCherry* |
|  | SAHT57-1 | *h^+^ leu1-32 ura4-D18 bqt1::LEU2 nuf2^+^::EGFP-ura4^+^ lys1^+^::sfi1^+^-mCherry aur1^+^::aur1^r^-Ptaz1-taz1∆myb-NLS-mCherry* |
|  | SKKT88-a | *h^—^ leu1-32 ura4-D18 rap1::ura4^+^ nuf2^+^::EGFP-ura4^+^ lys1^+^::sfi1^+^-mCherry aur1^+^::aur1^r^-Ptaz1-taz1∆myb-NLS-mCherry* |
|  | SKKT87-a | *h^+^ leu1-32 ura4-D18 rap1::ura4^+^ nuf2^+^::EGFP-ura4^+^ lys1^+^::sfi1^+^-mCherry aur1^+^::aur1^r^-Ptaz1-taz1∆myb-NLS-mCherry* |
|  | SAWT13-1 | *h^—^ ade6-M210 leu1-32 ura4-D18 taz1::ura4^+^ nuf2^+^::EGFP-ura4^+^ lys1^+^::sfi1^+^-mCherry aur1^+^::aur1^r^-Ptaz1-taz1∆myb-NLS-mCherry* |
|  | SAWT14-1 | *h^+^ ade6-M210 leu1-32 ura4-D18 taz1::ura4^+^ nuf2^+^::EGFP-ura4^+^ lys1^+^::sfi1^+^-mCherry aur1^+^::aur1^r^-Ptaz1-taz1∆myb-NLS-mCherry* |
|  | SAHT165-2 | *h^—^ leu1-32 ura4-D18 mto1::kan^r^ nuf2^+^::EGFP-ura4^+^ lys1^+^::sfi1^+^-mCherry aur1^+^::aur1^r^-Ptaz1-taz1∆myb-NLS-mCherry* |
|  | SAHT164-1 | *h^+^ leu1-32 ura4-D18 mto1::kan^r^ nuf2^+^::EGFP-ura4^+^ lys1^+^::sfi1^+^-mCherry aur1^+^::aur1^r^-Ptaz1-taz1∆myb-NLS-mCherry* |
|  | SAHT144-2 | *h^—^ leu1-32 ura4-D18 nuf2^+^::EGFP-ura4^+^ lys1^+^::sfi1^+^-mCherry aur1^+^::aur1^r^-Ptaz1- taz1∆myb-sad1-mCherry* |
|  | SAHT145-1 | *h^+^ leu1-32 ura4-D18 nuf2^+^::EGFP-ura4^+^ lys1^+^::sfi1^+^-mCherry aur1^+^::aur1^r^-Ptaz1- taz1∆myb-sad1-mCherry* |
|  | SAHT148-1 | *h^—^ leu1-32 ura4-D18 bqt1::LEU2 nuf2^+^::EGFP-ura4^+^ lys1^+^::sfi1^+^-mCherry aur1^+^::aur1^r^-Ptaz1-taz1∆myb-sad1-mCherry* |
|  | SAHT149-1 | *h^+^ leu1-32 ura4-D18 bqt1::LEU2 nuf2^+^::EGFP-ura4^+^ lys1^+^::sfi1^+^-mCherry aur1^+^::aur1^r^-Ptaz1-taz1∆myb-sad1-mCherry* |
|  | SKKT84-a | *h^—^ leu1-32 ura4-D18 rap1::ura4^+^ nuf2^+^::EGFP-ura4^+^ lys1^+^::sfi1^+^-mCherry aur1^+^::aur1^r^-Ptaz1-taz1∆myb-sad1-mCherry* |
|  | SKKT83-a | *h^+^ leu1-32 ura4-D18 rap1::ura4^+^ nuf2^+^::EGFP-ura4^+^ lys1^+^::sfi1^+^-mCherry aur1^+^::aur1^r^-Ptaz1-taz1∆myb-sad1-mCherry* |
|  | SAHT176-1 | *h^—^ ade6-M210 leu1-32 ura4-D18 taz1::ura4^+^ nuf2^+^::EGFP-ura4^+^ lys1^+^::sfi1^+^-mCherry aur1^+^::aur1^r^-Ptaz1-taz1∆myb-sad1-mCherry* |
|  | SAHT177-2 | *h^+^ ade6-M210 leu1-32 ura4-D18 taz1::ura4^+^ nuf2^+^::EGFP-ura4^+^ lys1^+^::sfi1^+^-mCherry aur1^+^::aur1^r^-Ptaz1-taz1∆myb-sad1-mCherry* |
|  | SAHT157-1 | *h^—^ leu1-32 ura4-D18 mto1::kan^r^ nuf2^+^::EGFP-ura4^+^ lys1^+^::sfi1^+^-mCherry aur1^+^::aur1^r^-Ptaz1-taz1∆myb-sad1-mCherry* |
|  | SAHT156-1 | *h^+^ leu1-32 ura4-D18 mto1::kan^r^ nuf2^+^::EGFP-ura4^+^ lys1^+^::sfi1^+^-mCherry aur1^+^::aur1^r^-Ptaz1-taz1∆myb-sad1-mCherry* |
| **8A, S5D, S5E** | SKKT190-2 | *h^–^ cnp1::nat^r^-Pcnp1-GFP-cnp1^+^ sfi1^+^::mCherry-nat^r^ lys1^+^::mat-P aur1^+^::aur1^r^-Ptaz1-taz1∆myb-sad1-mCherry* |
|  | SKKT188-1 | *h^–^ ura4-D18 nuf2^+^::EGFP-ura4^+^ sfi1^+^::mCherry-nat^r^ lys1^+^::mat-P aur1^+^::aur1^r^-Ptaz1-taz1∆myb-sad1-mCherry* |
|  | SKKT191-2 | *h^–^ cnp1::nat^r^-Pcnp1-GFP-cnp1^+^ sfi1^+^::mCherry-nat^r^ mto1::kan^r^ lys1^+^::mat-P aur1^+^::aur1^r^-Ptaz1-taz1∆myb-sad1-mCherry* |
|  | SKKT197-3 | *h^–^ ura4-D18 nuf2^+^::EGFP-ura4^+^ sfi1^+^::mCherry-nat^r^ mto1::kan^r^ lys1^+^::mat-P aur1^+^::aur1^r^-Ptaz1-taz1∆myb-sad1-mCherry* |
| **8B, S5D, S5E** | SKKT187-1 | *h^–^ leu1-32 cnp1::nat^r^-Pcnp1-GFP-cnp1^+^ sfi1^+^::mCherry-nat^r^ bqt1::LEU2 lys1^+^::mat-P aur1^+^::aur1^r^-Ptaz1-taz1∆myb-sad1-mCherry* |
|  | SKKT186-1 | *h^–^ leu1-32? ura4-D18 nuf2^+^::EGFP-ura4^+^ sfi1^+^::mCherry-nat^r^ bqt1::LEU2 lys1^+^::mat-P aur1^+^::aur1^r^-Ptaz1-taz1∆myb-sad1-mCherry* |
| **9A, 9B** | SKKT142-a | *h^—^ leu1-32 ura4-D18 sid4^+^::GFP-kan^r^ lys1^+^::Pnda3-mCherry-atb2^+^ cut11^+^::mCherry-nat^r(j)^* |
|  | SKK38-18D | *h^+^ leu1-32 ura4-D18 sid4^+^::GFP-kan^r^ lys1^+^::Pnda3-mCherry-atb2^+^ cut11^+^::mCherry-nat^r^* |
|  | SKKT144-a | *h^—^ ade6-M210 leu1-32 bqt1::LEU2 sid4^+^::GFP-kan^r^ lys1^+^::Pnda3-mCherry-atb2^+^ cut11^+^::mCherry-nat^r^* |
|  | SKKT143-a | *h^+^ ade6-M210 leu1-32 bqt1::LEU2 sid4^+^::GFP-kan^r^ lys1^+^::Pnda3-mCherry-atb2^+^ cut11^+^::mCherry-nat^r^* |
|  | SKKT145-a | *h^—^ ade6-M210 leu1-32 bqt1::LEU2 sid4^+^::GFP-kan^r^ lys1^+^::Pnda3-mCherry-atb2^+^ cut11^+^::mCherry-nat^r^ aur1^+^::aur1^r^-Ptaz1-taz1∆myb-sad1-mCherry* |
|  | SKKT162-a | *h^+^ ade6-M210 leu1-32 bqt1::LEU2 sid4^+^::GFP-kan^r^ lys1^+^::Pnda3-mCherry-atb2^+^ cut11^+^::mCherry-nat^r^ aur1^+^::aur1^r^-Ptaz1-taz1∆myb-sad1-mCherry* |
| **9B** | SKKT153-a | *h^+^ leu1-32 ura4-D18 sid4^+^::GFP-kan^r^ lys1^+^::Pnda3-mCherry-atb2^+^ cut11^+^::mCherry-nat^r^ aur1^+^::aur1^r^-Ptaz1-taz1∆myb-sad1-mCherry* |
|  | SKKT152-a | *h^—^ leu1-32 ura4-D18 sid4^+^::GFP-kan^r^ lys1^+^::Pnda3-mCherry-atb2^+^ cut11^+^::mCherry-nat^r^ aur1^+^::aur1^r^-Ptaz1-taz1∆myb-sad1-mCherry* |
|  | SKKT106-a | *h^—^ ade6-M210 leu1-32 ura4-D18 taz1::ura4^+^ sid4^+^::GFP-kan^r^ lys1^+^::Pnda3-mCherry-atb2^+^* |
|  | SKK38-18A | *h^+^ leu1-32 ura4-D18 taz1::ura4^+^ sid4^+^::GFP-kan^r^ lys1^+^::Pnda3-mCherry-atb2^+^ cut11^+^::mCherry-nat^r^* |
|  | SENT14-3 | *h^—^ ade6-M210 leu1-32 ura4-D18 taz1::ura4^+^ sid4^+^::GFP-kan^r^ lys1^+^::Pnda3-mCherry-atb2^+^ aur1^+^::aur1^r^-Ptaz1-taz1∆myb-sad1-mCherry* |
|  | SHM336-17C | *h^+^ leu1-32 ura4-D18 taz1::ura4^+^ sid4^+^::GFP-kan^r^ lys1^+^::Pnda3-mCherry-atb2^+^ cut11^+^::mCherry-nat^r^ aur1^+^::aur1^r^-Ptaz1-taz1∆myb-sad1-mCherry* |
| **S2** | SEN28-4A | *h^+^ leu1-32 ura4-D18 poz1::ura4^+^ cnp1::nat^r^-Pcnp1-GFP-cnp1^+^ lys1^+^::sfi1^+^-mCherry* |
|  | SEN28-7D | *h^–^ leu1-32 ura4-D18 poz1::ura4^+^ cnp1::nat^r^-Pcnp1-GFP-cnp1^+^ lys1^+^::sfi1^+^-mCherry* |
|  | SEN33-8C | *h^+^ ade6-M210 leu1-32 ura4-D18 poz1::ura4^+^ taz1::ura4^+^ cnp1::nat^r^-Pcnp1-GFP-cnp1^+^ lys1^+^::sfi1^+^-mCherry* |
|  | SEN33-9C | *h^–^ leu1-32 ura4-D18 poz1::ura4^+^ taz1::ura4^+^ cnp1::nat^r^-Pcnp1-GFP-cnp1^+^ lys1^+^::sfi1^+^-mCherry* |
|  | SKK33-6B | *h^–^ leu1-32 ura4-D18 dhc1-d3[::LEU2]^(r)^ cnp1::nat^r^-Pcnp1-GFP-cnp1^+^ lys1^+^::sfi1^+^-mCherry* |
|  | SKK33-19A | *h^+^ ade6-M216 leu1-32 ura4-D18 dhc1-d3[::LEU2] cnp1::nat^r^-Pcnp1-GFP-cnp1^+^ lys1^+^::sfi1^+^-mCherry* |

^a^This study; ^b^([Ding et al., 2004](#_ENREF_4)); ^c^([Nabeshima et al., 1998](#_ENREF_8)); ^d^([Chikashige et al., 2006](#_ENREF_2)); ^e^([Cooper et al., 1998](#_ENREF_3)); ^f^([Samejima et al., 2005](#_ENREF_10)); ^g^Yeast Genetic Resource Center; ^h^([Fujita et al., 2012](#_ENREF_5)); ^i^([Takayama et al., 2008](#_ENREF_11)); ^j^([Yoshida et al., 2013](#_ENREF_16)); ^k^([Nabetani et al., 2001](#_ENREF_9)); ^l^([Chikashige and Hiraoka, 2001](#_ENREF_1)); ^m^([Yamamoto and Hiraoka, 2003](#_ENREF_13)); ^n^([Lin and Smith, 1994](#_ENREF_7)); ^o^([Kitajima et al., 2004](#_ENREF_6)); ^p^([Yokobayashi and Watanabe, 2005](#_ENREF_15)); ^q^([Tomlin et al., 2002](#_ENREF_12)); ^r^([Yamamoto et al., 1999](#_ENREF_14)).

**References for S1 Table**

Chikashige, Y., and Y. Hiraoka. 2001. Telomere binding of the Rap1 protein is required for meiosis in fission yeast. *Curr Biol*. 11:1618-1623

Chikashige, Y., C. Tsutsumi, M. Yamane, K. Okamasa, T. Haraguchi, and Y. Hiraoka. 2006. Meiotic proteins bqt1 and bqt2 tether telomeres to form the bouquet arrangement of chromosomes. *Cell*. 125:59-69. doi:10.1016/j.cell.2006.01.048

Cooper, J.P., Y. Watanabe, and P. Nurse. 1998. Fission yeast Taz1 protein is required for meiotic telomere clustering and recombination. *Nature*. 392:828-831. doi:10.1038/33947

Ding, D.Q., A. Yamamoto, T. Haraguchi, and Y. Hiraoka. 2004. Dynamics of homologous chromosome pairing during meiotic prophase in fission yeast. *Dev Cell*. 6:329-341

Fujita, I., M. Tanaka, and J. Kanoh. 2012. Identification of the functional domains of the telomere protein Rap1 in Schizosaccharomyces pombe. *PLoS One*. 7:e49151. doi:10.1371/journal.pone.0049151

Kitajima, T.S., S.A. Kawashima, and Y. Watanabe. 2004. The conserved kinetochore protein shugoshin protects centromeric cohesion during meiosis. *Nature*. 427:510-517. doi:10.1038/nature02312

Lin, Y., and G.R. Smith. 1994. Transient, meiosis-induced expression of the rec6 and rec12 genes of Schizosaccharomyces pombe. *Genetics*. 136:769-779

Nabeshima, K., T. Nakagawa, A.F. Straight, A. Murray, Y. Chikashige, Y.M. Yamashita, Y. Hiraoka, and M. Yanagida. 1998. Dynamics of centromeres during metaphase-anaphase transition in fission yeast: Dis1 is implicated in force balance in metaphase bipolar spindle. *Mol Biol Cell*. 9:3211-3225

Nabetani, A., T. Koujin, C. Tsutsumi, T. Haraguchi, and Y. Hiraoka. 2001. A conserved protein, Nuf2, is implicated in connecting the centromere to the spindle during chromosome segregation: a link between the kinetochore function and the spindle checkpoint. *Chromosoma*. 110:322-334. doi:10.1007/s004120100153

Samejima, I., P.C. Lourenco, H.A. Snaith, and K.E. Sawin. 2005. Fission yeast mto2p regulates microtubule nucleation by the centrosomin-related protein mto1p. *Mol Biol Cell*. 16:3040-3051. doi:10.1091/mbc.E04-11-1003

Takayama, Y., H. Sato, S. Saitoh, Y. Ogiyama, F. Masuda, and K. Takahashi. 2008. Biphasic incorporation of centromeric histone CENP-A in fission yeast. *Mol Biol Cell*. 19:682-690. doi:10.1091/mbc.E07-05-0504

Tomlin, G.C., J.L. Morrell, and K.L. Gould. 2002. The spindle pole body protein Cdc11p links Sid4p to the fission yeast septation initiation network. *Mol Biol Cell*. 13:1203-1214. doi:10.1091/mbc.01-09-0455

Yamamoto, A., and Y. Hiraoka. 2003. Monopolar spindle attachment of sister chromatids is ensured by two distinct mechanisms at the first meiotic division in fission yeast. *Embo J*. 22:2284-2296. doi:10.1093/emboj/cdg222

Yamamoto, A., R.R. West, J.R. McIntosh, and Y. Hiraoka. 1999. A cytoplasmic dynein heavy chain is required for oscillatory nuclear movement of meiotic prophase and efficient meiotic recombination in fission yeast. *J Cell Biol*. 145:1233-1249

Yokobayashi, S., and Y. Watanabe. 2005. The kinetochore protein Moa1 enables cohesion-mediated monopolar attachment at meiosis I. *Cell*. 123:803-817. doi:10.1016/j.cell.2005.09.013

Yoshida, M., S. Katsuyama, K. Tateho, H. Nakamura, J. Miyoshi, T. Ohba, H. Matsuhara, F. Miki, K. Okazaki, T. Haraguchi, O. Niwa, Y. Hiraoka, and A. Yamamoto. 2013. Microtubule-organizing center formation at telomeres induces meiotic telomere clustering. *J Cell Biol*. 200:385-395. doi:10.1083/jcb.201207168
